# Multi‐Model Prediction of West Nile Virus Neuroinvasive Disease With Machine Learning for Identification of Important Regional Climatic Drivers

**DOI:** 10.1029/2023GH000906

**Published:** 2023-11-17

**Authors:** Karen M. Holcomb, J. Erin Staples, Randall J. Nett, Charles B. Beard, Lyle R. Petersen, Stanley G. Benjamin, Benjamin W. Green, Hunter Jones, Michael A. Johansson

**Affiliations:** ^1^ Global Systems Laboratory National Oceanic and Atmospheric Administration Boulder CO USA; ^2^ Now at Division of Vector‐Borne Diseases Centers for Disease Control and Prevention Fort Collins CO USA; ^3^ Division of Vector‐Borne Diseases Centers for Disease Control and Prevention Fort Collins CO USA; ^4^ Cooperative Institute for Research in Environmental Sciences University of Colorado Boulder Boulder CO USA; ^5^ Climate Prediction Office National Oceanic and Atmospheric Administration Silver Spring MD USA; ^6^ Division of Vector‐Borne Diseases Centers for Disease Control and Prevention San Juan PR USA

**Keywords:** West Nile virus, machine learning, prediction, climate, variable importance

## Abstract

West Nile virus (WNV) is the leading cause of mosquito‐borne illness in the continental United States (CONUS). Spatial heterogeneity in historical incidence, environmental factors, and complex ecology make prediction of spatiotemporal variation in WNV transmission challenging. Machine learning provides promising tools for identification of important variables in such situations. To predict annual WNV neuroinvasive disease (WNND) cases in CONUS (2015–2021), we fitted 10 probabilistic models with variation in complexity from naïve to machine learning algorithm and an ensemble. We made predictions in each of nine climate regions on a hexagonal grid and evaluated each model's predictive accuracy. Using the machine learning models (random forest and neural network), we identified the relative importance and variation in ranking of predictors (historical WNND cases, climate anomalies, human demographics, and land use) across regions. We found that historical WNND cases and population density were among the most important factors while anomalies in temperature and precipitation often had relatively low importance. While the relative performance of each model varied across climatic regions, the magnitude of difference between models was small. All models except the naïve model had non‐significant differences in performance relative to the baseline model (negative binomial model fit per hexagon). No model, including the ensemble or more complex machine learning models, outperformed models based on historical case counts on the hexagon or region level; these models are good forecasting benchmarks. Further work is needed to assess if predictive capacity can be improved beyond that of these historical baselines.

## Key Points

- Historical case counts and population density were the most important variables for predicting West Nile neuroinvasive disease cases
- No model, including machine learning models, significantly improved prediction beyond that of historical patterns in cases
- We found variability in the relative importance of climatic anomalies across models and regions, with generally low importance

## 1 Introduction

West Nile virus (WNV) is the leading cause of mosquito‐borne illness in the continental United States (Rosenberg et al., 2018). When humans are infected with WNV from the bite of an infectious mosquito, 75%–80% remain asymptomatic and approximately 20%–25% develop a self‐limiting febrile illness (e.g., symptoms including headache, body ache, nausea, vomiting, and rash) (Mostashari et al., 2001; Zou et al., 2010). However, in <1% of infections (1 in 150–244), the virus invades the central nervous system (neuroinvasive form), causing encephalitis, meningitis, and acute flaccid paralysis (Sejvar, 2007; Sejvar & Marfin, 2006). Of those with WNV neuroinvasive disease (WNND), approximately 10% die from infection and long‐term physical and mental sequalae are common in survivors (Sejvar, 2007). The incidence of WNND increases with age (Carson et al., 2012; McDonald et al., 2021). While WNND cases represent a small subset of all WNV infections, due to the severity of the disease, most persons with WNND are likely diagnosed and captured in national reporting systems (McDonald et al., 2021).

WNV was introduced into the United States in 1999 (Nash et al., 2001) and rapidly spread westward across the contiguous states, reaching the Pacific states in 2003–2004 (Kramer et al., 2019). Since then, WNV has become endemic with the highest average annual incidence (≥2 annual WNND cases per 100,000) generally in the Great Plains states (including MT, ND, SD, WY, CO, NE) (Lindsey et al., 2010; McDonald et al., 2021). Large numbers of WNND cases are also annually reported in cities like Los Angeles (CA), Phoenix (AZ), Chicago (IL), and Dallas (TX) (McDonald et al., 2021). During 1999–2021, 55,443 WNV disease cases (27,586 non‐neuroinvasive and 27,857 WNND) and 2,683 resulting deaths were reported to the CDC (Gould et al., 2023). WNND cases exhibit high spatial and temporal variability. During the endemic period for WNV (2005–2021), an average of 1,221 WNND cases were reported annually but ranged from 386 to 2,873 (Centers for Disease Control and Prevention, 2023). On average, 370 (12%) of the 3,108 counties in the continental US report WNND cases each year (range: 167%–693%, 5%–22%) (Centers for Disease Control and Prevention, 2023). Even in counties that have reported WNV cases, numbers can fluctuate dramatically year‐to‐year (e.g., Maricopa County, AZ reported 155 during 2019, 3 during 2020, and 1,487 during 2021) (Arizona Department of Health Services, 2023). Also, the median annual number of cases varies substantially across counties (e.g., reported medians <1 and >30).

The ecology of WNV is complex and exhibits spatial variation. The virus is maintained in enzootic transmission cycles between *Culex* mosquitoes and birds (primarily passerines) (Kilpatrick et al., 2007; McLean et al., 2001; Rochlin et al., 2019), but can spill over and cause disease in horses and humans, both of which are dead‐end hosts (i.e., do not infect biting mosquitoes) (Kramer et al., 2019). Four *Culex* mosquito species are the dominant vectors for WNV with geographic variation and overlap in regions where each has been implicated as the primary vector (Rochlin et al., 2019). Differences in the feeding preferences and breeding areas for the four species add to the complex ecology and make it challenging to compare one location to another. *Cx*. *pipiens* is the primary vector throughout the eastern United States as well as in urban areas of the northern Great Plains, Rocky Mountains, and Pacific Northwest. *Cx*. *restuans* is a primary vector in the east‐central US (Northeast, Mid‐Atlantic, and Central regions). Across the southeast and in urban areas of the southwest, *Cx*. *quinquefasciatus* acts as the dominant vector. In the more rural areas of the western United States, the primary vector is *Cx*. *tarsalis*. Passerine birds generally serve as enzootic reservoirs and amplification hosts (Kilpatrick et al., 2007; Komar et al., 2003). Hatch‐year birds often contribute to rapid viral amplification (Hamer et al., 2008).

Climate and weather factors like temperature and precipitation have a large impact on WNV transmission through modulation of mosquito and viral dynamics on relatively short timescales. Increasing temperature results in increased viral transmission rates and risk of zoonotic infection up to the thermal optimum (average temperature: 23.9–25.2°C (Shocket et al., 2020)), above which transmission decreases due to negative impacts on mosquito traits like survival. Below the thermal optimum, transmission increases as warmer temperatures increase mosquito development and biting rates (Ciota et al., 2014; Reisen, 1995) as well as viral replication rates while reducing the extrinsic incubation period (i.e., the time from when a mosquito is infected to when it becomes infectious) (Dohm et al., 2002; Kilpatrick et al., 2008; Reisen et al., 2006). Of note, extreme heat (e.g., above‐average‐temperatures and heat waves) often results in rapid increases in zoonotic transmission rate (Paz, 2015; Soverow et al., 2009). Increasing precipitation generally increases transmission rates through increased larval habitat (Landesman et al., 2007; Shaman et al., 2010, 2011), but intense precipitation events can wash out immature mosquitoes from these larval habitats (Gardner et al., 2012). The impact of precipitation anomalies in relation to human incidence of WNV varies across the United States with positive associations in the western United States and negative associations in the eastern United States (Hahn et al., 2015). In the West, increased precipitation may lead to an increased quantity of larval habitats for *Cx*. *tarsalis* across rural and agricultural areas while in the East, increased precipitation could wash immature *Cx*. *pipiens* mosquitoes from larval habitats like storm drains and sewers. Drought has also been found to be associated with increased WNV transmission, potentially by increasing bird‐mosquito contact at dwindling sources of water (Chase & Knight, 2003; Shaman et al., 2002). Overall, the most predictive environmental factors for WNV have been found to vary spatially (Hahn et al., 2015; Paull et al., 2017; Wimberly et al., 2014).

Transmission dynamics can be modulated at both short‐ and longer‐term timescales. The relatively short lifespan of adult mosquitoes during summer (∼10 days) enables rapid changes in mosquito dynamics and thus transmission rates in response to current climatic conditions. In contrast, passerine birds have longer lifespans (e.g., maximum reported lifespans are around 20 years with mean lifespans closer to 3–5 years (Klimkiewics & Futcher, 1998)). Additionally, birds that survive WNV infection develop lifelong antibodies to WNV (Fang & Reisen, 2006; Nemeth et al., 2009), resulting in longer‐term variation in contribution to WNV transmission. While weather anomalies are known to have multi‐faceted impacts on avian population biology (e.g., changes in demographic rates, phenology, distributions, and ranges) (Crick, 2004), little research has been done to link climatic impacts on avian populations and variation in WNV transmission dynamics.

Developing the capability to predict when and where WNV activity will occur would allow more timely and effective use of public health prevention activities (e.g., vector control, community messaging, and healthcare provider alerts). A wide range of models have been used to predict the geographic or temporal distribution of WNV in the United States (Barker, 2019; Reiner et al., 2013), including regression (e.g., Karki et al., 2020; Mori et al., 2018), compartmental models (e.g., Hartley et al., 2012), and Bayesian frameworks (e.g., Humphreys et al., 2021). Machine learning models have also been applied (e.g., Hess et al., 2018; Keyel et al., 2019), but have not been widely utilized. Most predictions of WNV were made on relatively small spatial scales and resolutions (i.e., city, county, or state) while some encompassed the contiguous United States. Predictions also generally derived from single models without comparison to baseline models. Models developed for one region often do not translate to others due to ecologic variation or availability of data sources between locations (Keyel et al., 2021). Ongoing WNV forecasting challenges, organized by the Centers for Disease Control and Prevention, aim to provide national and regional predictions of WNV activity yet, to‐date the best performing forecasts have leveraged only local historical incidence to make forecasts (Holcomb et al., 2023). To capture the complexities of WNV transmission and ecology, modelers often include parameters such as human demographics, reported human or veterinary cases, climate, hydrology, avian population dynamics, land use, and mosquito surveillance. However, the same set and resolution of variables are not consistently used across models so the relative importance of each cannot be assessed across multiple locations. The use of more complex models may allow for greater flexibility in identifying underlying relationships (e.g., climate and WNV relationships), but potentially at the expense of interpretability of relationships. Additionally, considering multiple individual models can build a more coherent understanding of the relationships by addressing different aspects of the multi‐factorial interactions. A systematic evaluation of county‐annual predictions of WNND cases from 15 models from the CDC's 2020 WNV Forecasting Challenge found that historical WNND cases was the strongest predictor of future case counts (Holcomb et al., 2023). Additionally, models that included climate or human demographic data were associated with better predictive performance while the inclusion of mosquito or land use data were associated with worse performance. Given the spatial heterogeneity in ecology, climate, and WNV incidence across the US, work is needed to identify regionally important factors to improve WNV prediction.

We performed a multi‐model analysis and evaluation of probabilistic predictions of WNND cases across the contiguous 48 states plus Washington DC (CONUS, 2015–2021). We made these annual predictions to assess predictive performance under an early warning framework. We used the machine learning models included in the multi‐model assemblage to identify region‐specific factors that were consistently important for prediction across years.

## 2 Materials and Methods

### 2.1 Study Area and Data

**Figure 1:**
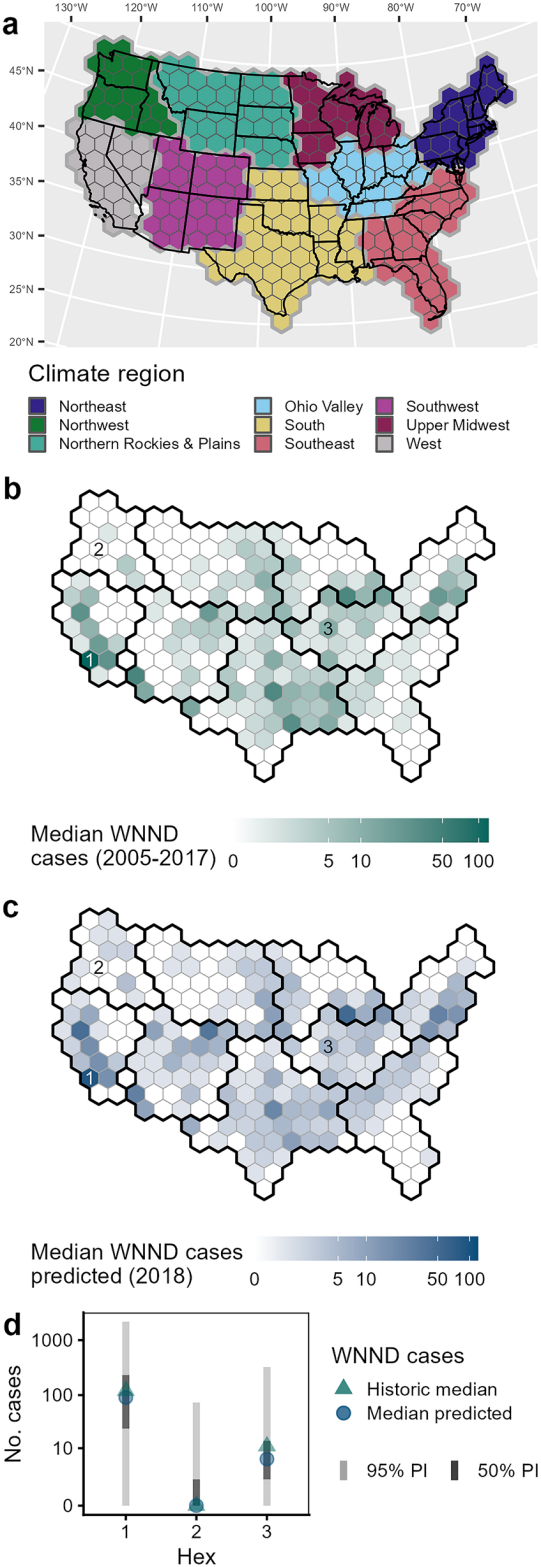
Study area and example model predictions. (a) Hexagonal grid used in modeling with climate regions indicated. State boundaries indicated for contextualization. The uncolored hexagon between the West and Southwest regions did not contain county‐level data and was not included in the analysis (see Section 2.1). (b) Median number of West Nile neuroinvasive disease (WNND) cases (2005–2017). (c) Median number of predicted WNND cases from an example forecast (prediction for 2018 from the neural network fit with seasonal climate data, see Section 2.2.2). Climate region outlines indicated in panels (b, c) use dark lines. (d) Historic median and median predicted WNND cases with 50% and 95% Prediction Interval for indicated hexagons in panels (b, c).

We predicted annual totals of WNND cases across CONUS using an equal‐area hexagonal grid (200 km diameter). We created the grid using an equal‐area projection of the CONUS counties (NAD83) and the *st_make_grid* function in sf package (version 1.0–8) (Pebesma, 2018; R Core Team, 2022). We used the 2020 U.S. Census TIGER/Line shapefile from the tigris R package (Walker, 2022). We divided the 263 hexagons into nine climate regions as defined by Karl and Koss (1984) (Figure 1a) to assess regional‐specific environmental, demographic, and disease‐specific factors. We used 21–48 hexagons per region as more than 50 hexagons per region was intractable for the random forest (RF) algorithm. We classified hexagons to a single climate region based on the largest spatial overlap with states included in each region (climate region boundaries defined by state boundaries). We chose to use a hexagonal grid rather than a geopolitical boundary (e.g., county) to remove biases due to wide variation in county sizes across the United States. Additionally, we chose a relatively large area (∼34,640 km^2^) for individual hexes (hexagons were larger than 3,103 counties (99.8%)) to align with our goal of a large‐scale, early warning framework for the predictive modeling.

We obtained annual totals of WNND cases from ArboNET, the national arboviral diseases surveillance system administered by the CDC (2005–2021, data for 2021 were provisional totals at the time of analysis) on the county scale (Centers for Disease Control and Prevention, 2023). We did not include case counts from the first 5 years after WNV was introduced into the United States (i.e., 1999–2004) as the invasion dynamics could be different from those during the endemic phase (2005–present). We converted these county totals to hexagon totals by assigning county WNND case counts to the hexagon which included the county centroid. Note that due to the large sizes of counties in the Western United States, one internal hexagon in the grid did not contain any county‐level data and thus was dropped from the analysis (Figure 1a).

We aggregated human demographic data to each hexagon using a similar procedure as with the WNND case data. Using county‐level demographic data (i.e., total population size, population >65‐year of age, and population density) from the 2010 U.S. Census (United States Census Bureau, 2021), we assigned information to a hexagon if the county's centroid was included within the hexagon. We calculated population density as the sum of the overlapping counties total population divided by the total area of all overlapping counties, that is, the average density for all included counties (people/km^2^).

We assigned climatic data to hexagons using data from the Parameter‐elevation Regressions on Independent Slopes Model (PRISM) data set (PRISM Climate Group & Oregon State University, 2022). We used monthly gridded (4‐km) data to calculate normalized anomalies (i.e., number of standard deviations away from the long‐term mean) in monthly and seasonal temperature (mean and minimum) and total precipitation per hexagon (2000–2021). We defined seasons as 3‐month groupings for winter (December–February), spring (March–May), summer (June–August), and fall (September–November). The date range allowed us to capture climatic conditions up to 5 years prior to the earliest included year of WNV data (2005) in order to investigate lagged effects of climate. We calculated normalized anomalies (number of standard deviations away from average) for each climatic variable relative to the 22‐year average since WNV was introduced into the United States (1999–2021). For all models with climatic data, we chose to use normalized anomalies instead of the raw temperature or precipitation amounts to reduce correlation among variables (see Text S1 in Supporting Information S1 for details).

To capture underlying mosquito habitats, we used land use classifications. Using the 2011 raster from the National Land Cover Database (30‐m resolution) (U.S. Geological Survey, 2014), we calculated the proportion of a hexagon that was urban (all “Developed” categories), crops (“Cultivated Crops” category), and wetlands (“Emergent Herbaceous Wetlands” category). Proportions were static across the study period.

### 2.2 Model Fitting and Selection

We fit a range of probabilistic models with variation in complexity to predict annual WNND cases by hexagon. Similar to a previous evaluation of WNND prediction in the US (Holcomb et al., 2023), we developed a series of baseline models (i.e., Always Absent naïve, negative binomial (NB), autoregressive, and autoregressive climate models) with increasing complexity to provide benchmarks in predictive performance when compared to the RF and neural network (NN) models (i.e., machine learning models). For each model type, we iteratively predicted hexagon‐level cases for 2015–2021 in each of the nine climate regions using data prior to each prediction year. For example, models were trained on data through 2014 to predict WNND cases in 2015. New models were then trained on data that included 2015 and were used to predict cases in 2016, and so forth. All models produced probabilistic predictions (see following sections and Text S1 in Supporting Information S1 for further details).

We also fitted a median ensemble using all forecasts except the Always Absent naïve model; we excluded this model because it was a very naïve model that had not performed well in previous WNV forecasting (Holcomb et al., 2023). To fit the ensemble, we used a linear opinion pool method (Jose et al., 2014; Stone, 1961) by taking the median probability assigned to predicted WNND case counts per hexagon and year across forecasts. This method of aggregation assumes that each forecast captures a possible outcome versus representing a noisy sample from a single distribution, thus maintaining between‐forecast uncertainty (Howerton et al., 2023).

#### 2.2.1 Baseline Benchmark Model

The Always Absent naïve model assigned all probability to the occurrence of zero cases. We fit a variety of NB models (NB‐hex, NB‐region, and NB‐nation) which incorporated historical case counts on different spatial scales, but the same temporal scale. The NB‐hex model fitted a NB model to historical cases from each individual hexagon independently and used this fitted distribution to generate probabilistic predictions of cases counts for the prediction year. We used Stan (rstan package (Stan Development Team, 2022)) to fit the distribution using Markov chain Monte Carlo sampling (see Text S1 in Supporting Information S1 for details). For the NB‐region model, we fitted a NB generalized linear model (GLM) (GLM; *glm*.*nb* function in the MASS package (Venables & Ripley, 2002)) to all historical cases in a climate region, using a categorical variable to indicate each hexagon in that region. Predictions for each hexagon thus included a hexagon‐specific mean and a region‐specific variance parameter. The NB‐nation model fitted a single NB distribution to historical case data from all hexagons in the United States, resulting in the same prediction for every hexagon in the country for each prediction year. As with the NB‐hex model, we fitted the distribution in a Bayesian context. Thus, NB‐hex uses only hexagon‐specific information, NB‐region uses hexagon and region information, and NB‐nation uses no hexagon‐specific information, providing performance benchmarks of models incorporating various degrees of spatial heterogeneity in case occurrence.

To incorporate temporal patterns of case counts, we also fitted an autoregressive model of order one (AR(1)) in each hexagon. As with the NB‐hex model, we fitted an AR(1) model (*Arima* function in the forecast R package (Hyndman & Khandakar, 2008; Hyndman et al., 2022)) to each hexagon using the hexagon‐specific logged historic case counts (ln(cases + 1)). To predict cases, we generated 1,000 bootstrapped samples by summing a sample from the predicted distribution (normal) and a randomly selected training residual. Then we fit a NB distribution using 23 quantiles (1%, 2.5%, 5%, 10%, 15%, …, 90%, 95%, 97.5%, 99%) of the bootstrap predictions to produce the probabilistic predictions. We considered two ways for converting bootstrap samples to a probabilistic prediction (see Text S1 in Supporting Information S1 for details). We chose the described method (bootstrap ‐>quantiles ‐>NB distribution) because the methods produced similar results and this method ensured a smooth distribution.

We developed a single AR(1)‐Climate model per region which consisted of an AR(1) model (fitted with ln(cases + 1)) with an exogenous seasonal climate covariate (see Text S1 in Supporting Information S1 for model selection details, Table S1 in Supporting Information S1 for the selected climate covariate). As with the AR(1) model, we predicted the distribution of cases by generating 1,000 bootstrapped samples (sum of sample from prediction and randomly selected training residual) and fitting a NB model to the quantiles of the bootstrapped samples.

#### 2.2.2 Machine Learning Models—Random Forests and Neural Networks

Both types of machine learning models used were fitted in a time series approach with lagged variables to capture the temporal sequence of these variables. Our final included variables were the following: lagged yearly WNND case counts, median annual WNND case counts from all previous years, monthly and seasonal climate (anomalies in mean temperature and total precipitation), population density, and land use (% urban, % crops, % wetlands). To capture long‐term trends in historical WNND case series, we included yearly cases lagged 1–5 years prior to the year of prediction. We also included the median annual reported WNND cases for all previous years to capture a not explicitly temporal metric of historical counts (e.g., median for 2005–2017 for predicting 2018, Figure 1b). To capture potential lagged effects on both bird and mosquito populations, we included climate variables lagged 1–5 years. Impacts on bird populations could be expected at the longer lag lengths (2 to 5‐year lags) while mosquito population impacts could be expected from the short lag lengths (1‐year lag). We also included climate data for the prediction year (0‐year lag) to capture within season climate impacts on mosquito population dynamics and viral transmission. Population density and land use variables were static in the models.

All WNND case data (predictors or outcome) were log transformed (i.e., ln(cases + 1)) for fitting both RF and NN models to improve fitting. To investigate the impact of different temporal resolutions of climate data on predictions, we fitted two versions of each type of machine learning algorithm, one with monthly climatic data and the other with seasonal climatic data (i.e., RF‐month, RF‐season, NN‐month, and NN‐season). As in other models, season were defined as 3‐month aggregations of climatic data (see Section 2.1). All other covariates were the same between models. Note that as with the above baseline models, we fitted a unique model in each climate region and for each prediction year. The following sections provide details on model tuning and fitting.

##### 2.2.2.1 Random Forest Models

We fitted RF models using the forecastML package in R (Redell, 2022), a package specific for time series prediction. For each prediction year (2015–2021), we used nested cross‐validation to leave out 2‐year “windows” of training data. Windows were non‐overlapping and, in the case of training periods with odd numbers of years, the final window consisted of a single year of data. We calculated in‐ and out‐of‐sample root mean squared error (RMSE) and mean absolute error (MAE) as well as pseudo‐*R* ^2^ for each model fit (i.e., for each model fit within the nested cross‐validation).

We investigated a range of hyperparameters (i.e., number of trees (*ntree*) and number of variables randomly selected to try at each node (*mtry*)) and selected the best value for each by comparing pseudo‐*R* ^2^ and out‐of‐bag RMSE and MAE across all prediction years. We assessed *ntree* as 10‐, 15‐, or 20‐times the number of input parameters and *mtry* as one‐third or one‐half of the number of input parameters.

The final RF models had 10‐times the number of trees as input parameters and, at each split, a random selection of one‐third of the number of input parameters were tried. Each tree was fitted using a randomly selected two‐thirds of the training data and the remaining one‐third of data used to calculate out‐of‐bag performance metrics (RMSE and MAE).

For the models fitted with the above selected hyperparameters, we generated probabilistic predictions per hexagon and year by fitting a NB distribution to quantiles of 1,000 bootstrapped samples, as in other models. For this, we summed a randomly selected prediction from one of the trained models from the cross‐validation loop and added a randomly selected training residual from any model fit in the nested cross‐validation for that hexagon (*calculate_intervals* function the forecastML package, (Redell, 2022)). Using the same 23 quantiles of bootstrapped samples as listed above, we fitted a NB distribution to predict the number of cases.

To quantify the influential variables (“importance”) in each region, we calculated permutation importance (Breiman, 2001a, 2001b; Fisher et al., 2019). Specifically, we randomly permuted each variable 10 times and calculated the mean percent change in prediction error (mean squared error [MSE]) across these permutations. We then calculated the median percent change in MSE for each predictor across trained models for each prediction year and across all prediction years.

##### 2.2.2.2 Neural Network Models

We were unable to identify similar R packages developed for NN time series analysis and therefore, developed a workflow similar to that of the RF algorithms. Using the neuralnet package in R (Fritsch et al., 2019), we iteratively fitted a unique model per climate region and prediction year. We used neural networks with a single hidden layer, resilient backpropagation with weight backtracking, learning rate factors of 0.5 (lower) and 1.2 (upper), and a maximum of 1 × 10^6^ iterations. We fitted 250 repetitions per model using randomly initialized connection weights, creating an ensemble of fitted models for each prediction. We calculated in‐ and out‐of‐sample RMSE and MAE for each model fit (i.e., for each model repetition).

We used the same set of lagged and static covariates as inputs to the neural networks as described above for the RF model. All variables were individually min‐max scaled for model fitting and prediction with unique scaling for each training period.

We selected appropriate hyperparameters (i.e., number of nodes in the hidden layer and activation function for the hidden and output layers) based on comparisons of a subset of individual models using combinations of the above hyperparameters. For this, we fitted unique models to predict 2015–2018 in each climate region and compared in‐ and out‐of‐sample RMSE and MAE, selecting the combination of hyperparameters that minimized out‐of‐sample RMSE and MAE. We tried networks with the number of hidden nodes equal to two‐thirds, one‐half, or the square root of the number of input nodes. We considered sigmoid and softplus activation functions for the hidden layer and linear and leaky rectified linear unit (with an alpha of 0.01) activation functions for the output layer. Thus, we used a cross‐validation scheme across unique models fit across different lengths of training data to select the final models. We used a subset (4 years) of the full period (7 years) for selecting the final model construction.

The final model had the following hyperparameters: the number of nodes in the hidden layer was equal to the square root of the number of input nodes, a sigmoid activation function on the hidden layer nodes, and a linear activation function on the output node.

Using the above selected combination of hyperparameters, we fitted neural networks for predictions for the full range of years (2015–2021). To generate probabilistic predictions per hex, we fitted NB distributions to 1,000 bootstrapped samples. To generate the bootstrap samples, we added the prediction from one randomly selected ensemble member and a randomly selected training residual from across all ensemble members for that hexagon. As before, we then fitted a NB model to the 23 quantiles of the bootstrapped samples. See Figures 1c and 1d for an example forecast.

To identify variable importance per region, we used Olden's method of summing connection weights per input variable (Olden & Jackson, 2002; Olden et al., 2004) using the NeuralNetTools R package (version 1.5.3) (Beck, 2018). For each model repetition, we calculated the relative magnitude (0–1 scale using absolute value) of the summed connection weights per predictor. We then calculated the median relative magnitude for each predictor across all 250 model repetitions per prediction year and region as well as across all model repetitions and prediction years. Similarly, we also calculated the median proportion of the summed connection weights per predictor that were positive (per year and for all years) to capture the overall direction of effect for each predictor on the number of predicted WNND cases.

### 2.3 Model Comparison and Evaluations

We evaluated model skill and compared performance across models per climate region using logarithmic scores (Gneiting & Raftery, 2007; Rosenfeld et al., 2012). A logarithmic score is a proper scoring rule based on the predicted probabilities in relation to the observed case counts. It is calculated as log(*p*_*i*_) where *p*_*i*_ is the probability assigned to the observed outcome. For each model, we calculated the probability assigned to the observed number of cases using the fitted NB distribution for each hexagon and year. We compared mean logarithmic score per year and for all years per region to rank model performances. For calculating mean score, we truncated individual logarithmic scores to −10 to prevent scores of negative infinity which would occur with zero probabilities (i.e., complete misses in prediction).

To compare the performance of each model type across regions and given a specific set of variables potentially related to forecasts skill (historical and observed case counts), we used Bayesian GLMs. For the analyses, we converted the logarithmic scores to surprisal values by changing the sign (−log(*p*_*i*_)). Thus, we transformed the outcome to a continuous positive value that could be approximated by a Gamma distribution and was on the same scale as the original logarithmic scores. For logarithmic scores of zero (i.e., perfect forecasts), we converted these to a small non‐zero value (1 × 10^−10^) between zero and the next smallest score (1.53 × 10^−9^) as zero is undefined in Gamma distributions. This truncation and the truncation of logarithmic scores to −10 introduced artificial densities at the corresponding surprisal values. However, the Bayesian model interprets these observations as uncertain and the Markov Chain Monte Carlo sampling distributed these observations across a wider range of values.

We used Gamma distributed (log link) Bayesian GLMs fitted with Stan, using the *stan_glm* function in the rstanarm package (version 2.21.3) (Brilleman et al., 2018; Goodrich et al., 2022), to assess relative performance of models overall and given historical and observed WNND case counts. We omitted the Always Absent model from regression analyses as it was a naïve model that did not include any historical case data. For each of the following three regressions, we ran four chains with a burn‐in of 500 samples, and then collected an additional 1,500, resulting in 6,000 samples across the four chains. We used the default, weakly informative normal priors. We checked all models for convergence. For the first model, we regressed a categorical variable for each of the 10 models against surprisal of each hexagon and year. To identify differences in model prediction overall, we calculated the difference in estimated marginal means (emmeans R package (Lenth, 2023)) for all pairwise comparisons of included models, estimating the median and 95% highest probability density of the difference in surprisal. Building this first model, the second model added climate region (main effect and interaction with model type). We calculated the difference in estimated marginal means for all pairwise comparisons of models per region. For the third model, we added covariates to assess variation in skill attributed to the history of WNND cases and deviations in the observed case counts relative to the history. We dichotomized historical case counts per hexagon and year (*histCases*) as ever reported a case (i.e., ≥1 total case from 2005 to the year prior to the prediction year) or never reporting a case (i.e., zero total cases in this period). Similarly, we dichotomized the number of observed WNND cases in the predicted year into zero cases or ≥1 case (*ObsCases*). For the regression, we included the logged number of observed case count (ln(cases + 1)), model, *histCases*, and *ObsCases* as predictors. We included the main effect of logged cases in addition to the main effects, all double interactions, and the triple interaction of model, *histCases*, and *ObsCases*. We estimated the median and 95% Prediction Interval (PI) of the surprisal for each model.

To investigate the impact of using normalized climate anomalies on estimated variable importance and predictive performance, we fitted an additional machine learning model. We selected the best machine learning model (i.e., the one with the lowest logarithmic score across all regions) and re‐fit it using raw values for all climate covariates; all other covariates and model selection procedures remained the same. We compared variable importance and mean logarithmic score between this model and the one fitted with the corresponding climate anomalies.

## 3 Results

### 3.1 Regional Performance of Models

**Figure 2:**
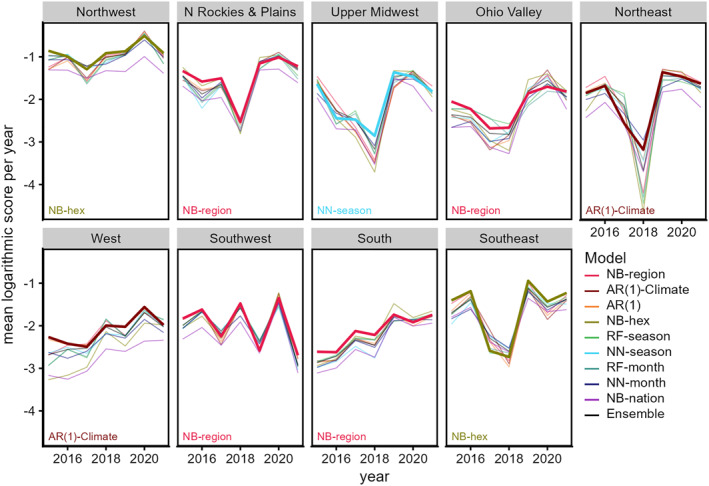
Mean logarithmic score of models per year for West Nile neuroinvasive disease prediction. Bold lines and text indicate the model with smallest mean logarithmic score overall for each region (“best” model). The Always Absent model was omitted due to scale (see Figure S3 in Supporting Information S1). The panels are ordered by relative geographic location of the nine climate regions (see Figure 1a).

Mean logarithmic scores from each model generally had more variability across regions and years than between models (Figure 2). The Always Absent model consistently scored worse than all other models (Figure S3 in Supporting Information S1). Among the other models, relative performance varied by year and the rank of models varied between climate regions. In six regions (Northwest, Northern Rockies & Plains, Ohio Valley, Southwest, South, and Southeast), either the region‐ or hex‐based NB model ranked first. In the Northeast and West, the AR(1)‐Climate model ranked first. The NB‐nation model ranked near the bottom, often with the RF and NN models. However, in the Upper Midwest regions, the seasonal NN model ranked as the top model. For both kinds of machine learning algorithms, models that used seasonal climate variables tended to have higher skill than those that included monthly climate variables. The ensemble ranked second to sixth across climate regions. In each region, most of the models had similar skill as the baseline NB‐hex model (Figure S4 in Supporting Information S1) except the Always Absent model which had worse skill (Table S2 in Supporting Information S1).

### 3.2 Performance of Models Across the Contiguous United States

**Figure 3:**
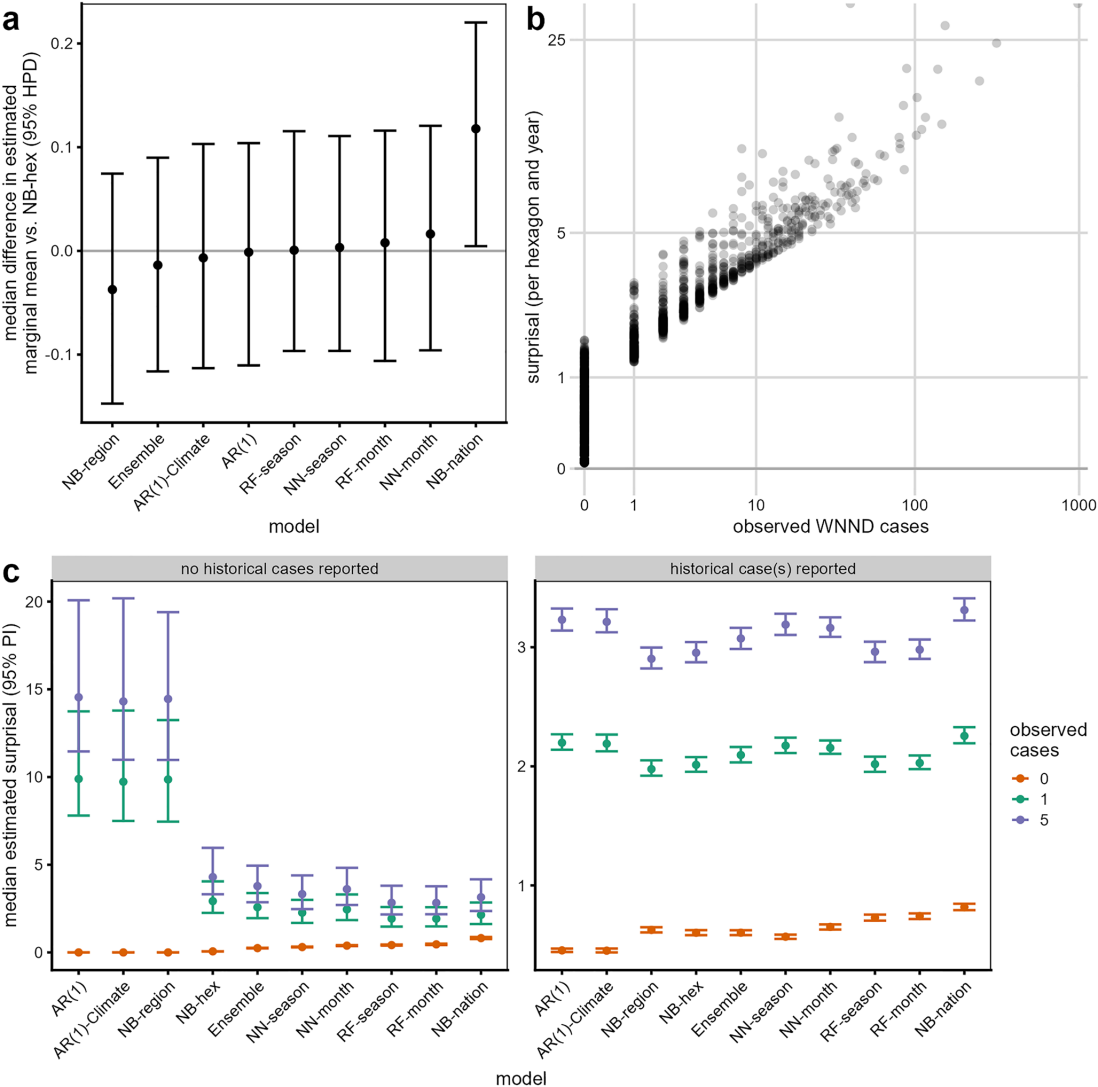
Predicted forecast skill of models. (a) Difference in estimated marginal means of predicted surprisal for models relative to the NB‐hex model (see Table S3 in Supporting Information S1). Increasing values of surprisal indicate worse forecast skill. (b) surprisal from the NB‐hex model per hexagon and year relative to the reported number of cases. Similar relationships were observed for all models. (c) Median estimated surprisal (95% Prediction Interval) for models when accounting for the reported number of cases and whether any cases had been previously reported in a hexagon. See Section 2.3 for details on regressions.

Across the regions, most models had similar skill to the baseline NB‐hex model (Figure 3a), except for the NB‐nation model and the Always Absent model which had even worse performance (Table S3 in Supporting Information S1). While the Always Absent model was worse than all other models, the NB‐nation model was worse than all models except the NN‐month model.

For all models, predicted surprisal was highly associated with the number of cases reported, reflecting sensitivity of the surprisal to that number (example from NB‐hex in Figure 3b). Accounting for the observed number of cases and whether any cases had been previously reported in a hexagon, we found a wider range of expected forecast skill across models in locations with no historical cases (Figure 3c). The NB‐hex, NB‐region, and autoregressive models performed better for these locations when compared to the machine learning models when no cases were observed. However, when at least one case was observed, the machine learning models were predicted to have higher skill, with the performance of the NB‐region and both autoregressive models predicted to be much worse. In hexagons where previous cases had been reported, we estimated smaller variation in median surprisal per model with increasing numbers of observed cases. In general, the machine learning models fitted with seasonal climatic anomalies had better expected skill than those fitted with monthly climatic anomalies.

### 3.3 Importance of Variables in WNND Prediction

The NB models and the AR(1) model used only historical WNV data for the specified locations, while other models were able to leverage climate data. Using single climate variables to assess AR(1)‐Climate models, we found high variability in predictive performance over years and regions (Figure S1 in Supporting Information S1), with spring and winter conditions for the current or previous year often being the best predictors (Table S1 in Supporting Information S1). Analyzing the hexagon‐specific coefficient estimates within each region we found high variability in both strength and direction for both the autoregressive component and the selected climate variable, with some hexagons having much stronger associations than others with the climate variable (Figure S2 in Supporting Information S1).

**Figure 4:**
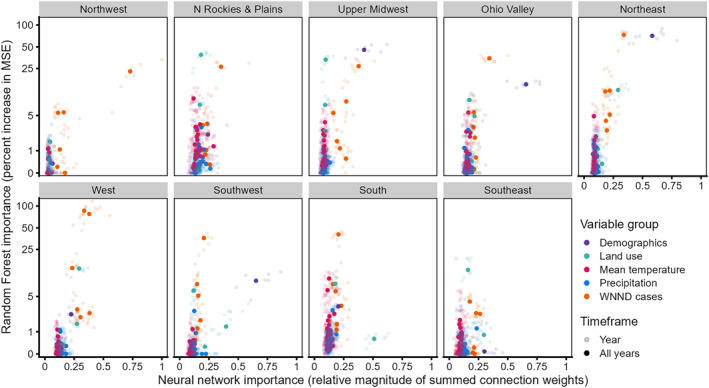
Variable importance for machine learning models fit with normalized anomalies in seasonal climatic data. Random forest importance (*y*‐axes) measured as median percent increase in mean squared prediction error when variable permuted. Neural network (*x*‐axes) importance measured as median relative magnitude of summed connection weights. See Figure 5 and Table S5 in Supporting Information S1 for further details on variable importance. Order of panels represent the relative geographic location of the nine climate regions in the US (see Figure 1a).

**Figure 5:**
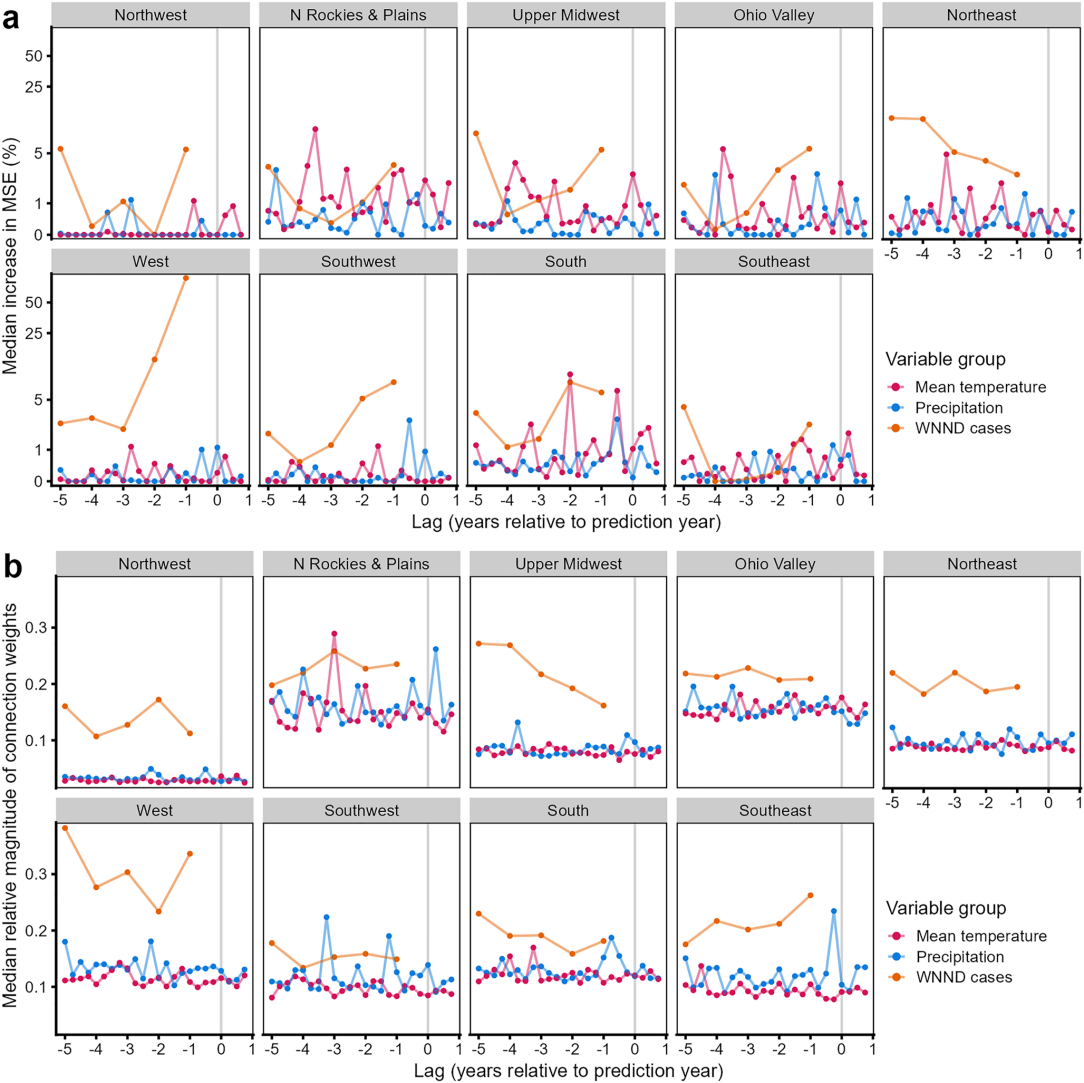
Median importance of lagged variables in models fitted with normalized anomalies in seasonal climate data. Importance based on (a) median increase in prediction error (mean squared error) when variable permuted in random forest models or (b) median relative magnitude of summed connection weights in neural network models. Importance measured across all models and prediction years. Lags of 0‐years indicate values from prediction year. See Table S5 in Supporting Information S1 for further details on variable importance. Order of panels represent the relative geographic location of the nine climate regions (see Figure 1a).

For the RF and NN models, median or lagged historical WNND case counts consistently had high importance across climate regions for models fit with normalized anomalies in seasonal (Figure 4) or monthly (Figure S5 in Supporting Information S1) climate data. Population density also often ranked high. In contrast, climatic variables generally had relatively lower importance, with both current (i.e., prediction year) and lagged anomalies in mean temperature often in the lowest ranking (see Figure 5 and Figure S6 in Supporting Information S1). Land use variables (urban, crops, and wetlands) had a much wider variation in importance across regions. Additionally, we observed consistency in the magnitude of a variable's median importance for each prediction year and with the importance across all years (Figure 4 and Figure S5 in Supporting Information S1).

We found higher concordance in the top‐ranking variables for seasonal versus monthly versions of the same model than we did between the RF and NN models (Table S5 in Supporting Information S1), as well as variation between which variables were selected per climate region. In most regions, there was disagreement on the type of climate anomaly, lag, and timing within the year (Table S5 in Supporting Information S1). For example, in the Northern Rockies and Plains region, the most important climate variable in the RF‐season model was the summer mean temperature anomaly (4‐year lag), in the RF‐month model, the mean temperature anomaly for July (1‐year lag), and in the NN‐season model, the winter mean temperature anomaly (3‐year lag). No climate variables were in the top six for any region for the NN‐month model.

For NN models, which enable assessment of importance and direction of effects, the median direction of effects often exhibited a reversed “C” shape relative to median rank (Figure S7 in Supporting Information S1). In other words, variables with higher importance often had a clear positive or negative effect (proportion of positive summed connection weights across model repetitions near 1 or 0) while variables with lower rank had low agreement in direction across model repetitions (proportions near 0.5). In general, lagged WNND case variables had both high rank and positive effects, but some had inconsistent estimates of the direction of effect (i.e., proportions near 0.5). Demographics and land use variables were fairly consistent in relative rank across seasonal and monthly models for each region. Higher population density was associated with increased risk except for in the West and Southeast regions. Urban land use always had a positive effect while crops and wetlands had positive effects in 6 of 9 regions each. The Northern Rockies and Plains and West had no strong association with either crops or wetlands, while the Southeast had a positive association with only wetlands and the Southwest with only crops. Across regions, precipitation (seasonal and monthly aggregations) had mid‐range importance with both positive and negative directions of effect and temperature generally had low rank and summed connection weights close to 0.5.

We compared the importance and predictive performance of the RF models fit with either seasonal climate anomalies or the raw values (see Text S1 in Supporting Information S1 for details). Broadly, we found similar ranking of variables between the model versions (Figure S8 in Supporting Information S1). Precipitation variables had somewhat higher importance when included as raw totals versus normalized anomalies. In contrast, lagged WNND cases tended to have somewhat higher importance when climate anomalies were included. However, we estimated very similar mean logarithmic scores (Table S4 in Supporting Information S1), indicating similar predictive accuracy of the two model versions.

## 4 Discussion

Actionable, early warning predictions of periods of high‐risk for zoonotic WNV transmission would be extremely valuable for public health and vector control agencies across the United States. Additionally, identification of regionally specific important predictors could tailor prediction and prevention activities. We utilized a multi‐model framework for predicting annual WNND cases in different climate regions with machine learning models to assess predictive accuracy and identify highly influential variables. Given the attributed importance of climate for WNV, we expected to improve forecasts with regional climate data and machine learning models. Surprisingly, we found similar predictive skill across a range of model complexities and covariate inclusions, with the history of cases consistently identified as highly influential. Population density also had high importance across several climate regions. In contrast, we did not find consistency in the climate variables (normalized anomalies) identified as important within regions, and overall, these variables did not improve predictive performance.

In general, we did not find any significant difference in skill between benchmark models based on historical WNV data alone and machine learning models fitted with demographic, land use, and climate data. Two models were significantly worse than others, the NB‐nation baseline and the naïve Always Absent models. The Always Absent model does not use any prior information on reported WNND cases while the NB‐nation model considered all historical WNND case data but no geographical information. The poor performance of these models compared to models with hexagon‐specific information indicates the importance of local case data to improve predictions. For the other nine models, which used hexagon‐specific data, rankings based on mean logarithmic score varied between region, but the magnitude of the differences was small, leading to very similar predicted skill across regions. However, the resulting skill of these models indicates there may still be room for improvement in predictive capacity of WNND cases, but the upper limit on achievable skill (i.e., a “perfect” forecast) is currently unknown.

Among the suite of better‐performing models were three baseline models relying only on historical case data: NB‐region, NB‐hex, and AR(1). In a previous, collaborative WNND forecasting project (Holcomb et al., 2023), a NB model equivalent to the NB‐hex model was found to be one of the best predictors of county‐level WNND cases in the next year, a slightly different geographical scale but a very similar result to what we found here. Previous forecasting efforts for other infectious diseases (Biggerstaff et al., 2018; Cramer et al., 2022; Johansson et al., 2019; Reich, Brooks, et al., 2019; Reich, McGowan, et al., 2019) have demonstrated that ensembles produce robust forecasts by balancing strengths and uncertainty from diverse models. While we replicated this overall finding here, with the ensemble ranked second to sixth in individual climate regions, we found that even the ensemble was unable to outperform predictions based on historical patterns, unlike for seasonal influenza (Lutz et al., 2019). One reason for this could be that the underlying assumptions of our models were not diverse enough to fully leverage the power of an ensemble.

While the overall skill for nine of the 10 models was similar, regression analyses identified specific differences in predicted skill based on historical case counts and observed case counts that provide insight on forecast failures. For all predictions, we found a general association between higher observed values and increased surprisal (worse skill) as has been noted in other forecasting studies (Bosse et al., 2022). Accounting for this relationship, we found important between‐model differences in skill for different scenarios. Notably, despite performing relatively well overall, the NB‐region, AR(1)‐Climate, and AR(1) models all had very poor estimated skill for hexagons reporting cases for the first time (i.e., no historical cases and at least one case reported in the forecasted year). These models were generally more confident about predicting zero cases, which led to better scores in most locations but also these occasionally very bad scores. This effect was most pronounced in the two AR(1) models which also show high divergence in hexagons with previously reported cases. The NB‐nation model provides an example of the opposite end of the spectrum; it makes the same prediction for all hexagons and thus has a lower probability of zero cases, despite zero cases being the most common outcome. The NB‐nation scores were similar to other models whenever cases were reported, but were the worst, on average, when no cases were reported. The NB‐hex model likely performed better than the other benchmark models because it used local data (unlike NB‐nation) and considered that only a limited number of previous years had been observed, thus having less confidence about a zero prediction. The machine learning models also showed more balanced performance, indicating that they also incorporated more uncertainty, giving non‐zero case counts some weight, even when none had previously been observed. Across hexagon‐years, 47.3% (2,116 of 4,471) were zeros and only 10 were first detections. Thus, overall performance metrics reflect mostly locations with previous cases or those with no previous cases and no current cases. However, better understanding where emergence is likely and unlikely is critical for public health and the models assessed here all exhibited trade‐offs in this aspect.

All the machine learning models identified variables related to local WNV history as highly influential for all climate regions. Median historic case counts ranked as the most important or one of the top six most important variables in all regions and machine learning model types with 1‐ and 5‐year lagged case counts also often highly ranked. The importance of 5‐year lags could suggest some influence of changes in immunity levels in avian populations, but further work is needed. The relative magnitude of importance of various lags for case counts varied across regions. In the NN models, some of these historic case variables (often 2‐ to 4‐year lags) had proportions of positive connection weights near 0.5, indicating disagreement on the direction of effect across individual model repetitions. While it is possible that these indicate trends in WNV transmission beyond simple annual cycles, the uncertainty in the direction of the impact could also reflect collinearity with other variables and lags. Regardless of that uncertainty, the machine learning models, like the NB and AR(1) models, all point to historical patterns as the most reliable known predictor of future WNND cases.

Higher population density was also often identified as an important indicator of increased predicted WNND risk. This variable ranked first or second in importance for both RF and NN models in the Ohio Valley, Upper Midwest, and Northeast climate regions. While the exact reason for its importance is unclear, this variable could reflect a higher density of human‐mosquito interactions. It may also help identify more urbanized areas that have habitat for *Cx*. *pipiens* and *Cx*. *restuans*, the dominant WNV vectors in the climate regions where it was most important (Rochlin et al., 2019). However, the underlying mosquito distributions may not fully explain the mechanism for the importance of population density across climate regions. In the West and Northwest, population density was not ranked highly while *Cx*. *pipiens* complex mosquitoes have been implicated as dominant vectors in urban areas in these regions. In the Southwest, population density also ranked high in both NN and RF models where different vectors have been implicated as dominant in urban and rural settings, *Cx*. *pipiens* complex and *Cx*. *tarsalis*, respectively (Rochlin et al., 2019). Other urban‐rural differences, like access to healthcare or age structure, not explicitly captured in our analysis, could also play a role. Differences in the underlying algorithms for RF and NN models may be capturing different aspects of the nonlinear relationships among variables, providing evidence that multiple groupings of variables (like demographics and land cover), each capturing a different part of the complex dynamics, can have similar predictive power.

Land cover variables were often ranked as important variables in regions, but we did not find consistency in which land cover variables were identified across machine learning model types within and between each region. For both machine learning model types, we found consistency in the order and relative magnitude of the importance of land use variables across models fitted with seasonal or monthly climate data. However, there were limited regions (West, Northeast, and Ohio Valley) where the same land use variable (urban) was identified in the top six variables by both a RF and a NN model. Variation in the estimated importance of these variables across modeling types could reflect differences in the underlying machine learning algorithm and that multiple, inter‐related factors are contributing to WNV transmission ecology. Bowden et al. (2011) identified a positive association of WNND cases with urbanized land cover across the northeastern US (i.e., generally the Ohio Valley, Northeast, Upper Midwest, and Southeast climate regions) and a positive association with crops or grasslands in the western US (i.e., the Northwest, West, Southwest, Northern Rockies and Plains, and South climate regions). The authors attributed these results to the broad distributions of dominant vectors; in the eastern US, *Cx*. *pipiens* lay eggs in more urban larval sites (e.g., storm drains and sewers) while in the west, *Cx*. *tarsalis* tend to use agricultural areas for larval breeding sites (Reisen, 2012; Reisen & Reeves, 1990). However, the authors also identified a positive association of WNND cases and crops in more eastern areas of the United States (i.e., the Southeastern, Ohio Valley, and Northeastern climate regions) where *Cx*. *tarsalis* are not generally found (Gorris et al., 2021), indicating that the associations with land cover are not exclusively related to vector distributions. Also, differences in management practices (e.g., use of flood irrigation) may exist across CONUS, contributing to regional variation in land cover importance. As our machine learning models incorporated a relatively large number of factors, we may not have identified land cover categories as important because we potentially captured other, underlying influential factors on WNV transmission.

We did not identify any climatic factors with consistent high importance for WNND prediction within climate regions. We found a general agreement on the relative ranking of climate variables within a region and between season and month versions, but importance was not consistent between RF and NN models. Models with seasonally summarized climate variables often had better skill than versions with monthly variables, but the differences in prediction were not substantive. Additionally, the top climate covariates in the RF and NN models were not consistent with those selected for the AR(1)‐Climate models. While the exogenous covariates for the AR(1)‐Climate models were selected from a somewhat different, yet overlapping set of candidate variables, we would expect to see similar types (i.e., temperature or precipitation anomalies) selected across models due to the attributed importance of climate to accurate WNV prediction (DeFelice et al., 2018; Hahn et al., 2015; Hess et al., 2018; Landesman et al., 2007; Shaman et al., 2010, 2011; Wimberly et al., 2022). In a few instances, similar climate anomalies (i.e., temperature or precipitation) were identified by the AR(1)‐Climate and machine learning models, but often with different lags. For example, in the West, Hahn et al. (2015) identified anomalies in winter mean temperature and spring precipitation as significant factors. Here, spring mean temperature was selected for the AR(1)‐Climate model here and no climate variables were among the most important in the RF or NN models. Interestingly, the AR(1)‐Climate model had the best skill overall in the West region, but performance was not vastly different than other models.

The inconsistencies in selected climate variables across models within each climate region points to further evidence that there is not a single or even set of climate factors and lags that is consistently highly predictive of annual WNND cases. Additionally, the performance of both machine learning and AR(1)‐Climate models per region were very similar with little consistency in rankings across prediction years; no single seasonal temperature or precipitation variable was consistently predictive. Thus, while climate factors can modulate WNV transmission, climate variables are not the only driver and the relative influence of climate likely can change between years and locations due to the multi‐faceted ecology of WNV. Previous improvements in WNV prediction with climate have been achieved on smaller spatial and temporal scales (Davis et al., 2018; DeFelice et al., 2018; Hess et al., 2018; Keyel et al., 2019; Wimberly et al., 2022). We attempted to develop local forecasts within climate regions (i.e., 200 km diameter hexagons) where other ecological factors are likely more consistent, but WNV dynamics may be consistent only at even smaller geographical scales necessitating even more flexibility. Moreover, climatic and other factors found to be predictive on a fine scale may not hold at coarser scales (Cohen et al., 2016).

We also found complex results relative to the potential timing of the relationship between climate and WNV transmission. We included lags of up to 5 years to allow for potential long‐term impacts on mosquitos or avian hosts, but the top climate variables were generally had lags of 0–2 years. Across lags, the importance of climate variables remained low. This finding is of particular interest for zero lags as it implies that even if we had climate data for the coming summer ahead of time (which in practice is not possible, except as a forecast), we would not be able to substantially improve WNND forecasts relative to forecasts made using climate from longer lags. In contrast, lagged historical cases up to 5 years often had consistently high importance (rank and relative magnitude). Previous studies using distributed lags (up to 36 months) of meteorological conditions (e.g., temperature, precipitation, and drought) similarly found significant lagged effects on WNND cases at the county scale, but did not consider lags up to 5 years (Davis et al., 2018; Smith et al., 2020; Wimberly et al., 2022). However, none of these models were compared to a historical baseline, like a NB model, to assess relative skill. Climate anomalies on relatively long lags may still impact the occurrence of WNND cases, but potentially their impact is not as apparent; the effect of previous conditions may be encapsulated in the historical case counts. Given the strong performance of models just based on historical case counts, work is needed to disentangle the relative contributions of previous conditions (e.g., climate anomalies, environmental conditions, and demographics) and changes in conditions over time (e.g., changes in land use or demographics) on observed historical counts to improve predictions.

At the regional level, we identified groups of variables and modeling approaches that were associated with improved skill. Across all regions, historical data were important, but particularly in the Northwest region, where this variable was uniquely highly important in both RF and NN models. In the Ohio Valley and Northeast regions, population density was also important. In the West, Northern Rockies and Plains, South, and Southeast regions, both historical case data and land cover (urban or crops for the West; urban for Northern Rockies; wetlands for the South; and urban or wetlands for the Southeast) ranked highly. In the Southwest and Upper Midwest regions, historical cases, population density, and a landcover (crops for the Southwest and urban for the Upper Midwest) were important. While these variable groups might inform further investigation and improved prediction of regional WNV transmission, we did not find clear patterns between highly important variables and best scoring modeling approaches. Rather, the majority of models had similar performance in each region with variation in their relative order. A machine learning model performed best in only a single climate region (the Upper Midwest). Overall, the NB‐hex and NB‐region consistently performed near the top and should therefore be good benchmark models for regional and sub‐regional (e.g., hexagon) prediction.

A strength of our approach was to use a multi‐model framework to evaluate a variety of model constructions with increasing complexity in structure and covariate inclusion using a single standardized performance metric. Additionally, we fitted each of the machine learning models using the same data set so we can attribute differences in performance to algorithms and not data availability. Variable importance identified by the RF and NN models can guide the development of future models including more sophisticated machine learning model architectures like long short‐term memory networks (Hochreiter & Schmidhuber, 1997) and boosted regression trees (Friedman, 2001); types of models that could improve predictive performance.

Our aggregation of covariates from various finer scale resolutions to the hexagon scale for analysis might not have captured the underlying fine‐scale relationships of these variables. For example, our choice of using the PRISM data set (4‐km resolution) to calculate climatic anomalies may not have fully captured the local conditions to which mosquitoes and birds were exposed. Also, our assessment of potential mosquito habitat (i.e., land cover) used a finer resolution (30‐m resolution) and there was no implicit connection with climate to capture the differential conditions in mosquito breeding habitats. Thus, the relatively large hexagons we chose might not reflect these small‐scale variations, making the model sensitive to hexagon size. However, at the relatively large spatial and temporal scale of models, these underlying associations may not be essential to describe the associations in our modeling framework and the large‐scale patterns of climate and land cover are consistent across hexagons. On the other hand, WNND case data were available at the county level, a much coarser scale than the environmental covariates, limiting the hexagon size. Reducing hexagon size could have led to hexagons without cases data, especially in the West due to large county sizes, removing potentially influential portions of the county from analysis. Any data set inherently introduces some bias, and the choice should be dependent on the intended application (Alexander et al., 2020; Daly et al., 2008). Further research could be done to evaluate the relative impact of different data sources and spatial scale on resulting prediction of infectious diseases as well as attribution of associated factors.

Large‐scale probabilistic early warning predictions like those in our modeling framework may be useful to mobilize limited public health resources to address predicted burden of disease. However, finer scale predictions (i.e., weekly neighborhood scale) could be extremely useful for within season decisions by public health and vector control agencies (e.g., rapid targeting local areas of developing high‐risk).

## 5 Conclusions

Human infection with WNV results from the complex interplay of mosquito vectors, avian hosts, human populations, and the environment. Using a multi‐model framework (including machine learning models) with a consistent set of covariates to assess prediction of annual cases of WNND on a hexagonal grid within climate regions of CONUS, we identified region‐specific factors that were consistently important for prediction. We found that historical numbers of cases were the most reliable indicator for forecasting future WNND cases regardless of the modeling approach. Models using local WNND case data performed as well or better than more complex machine learning models with more data as well as an ensemble of all included models. In addition to lagged case counts, machine learning models identified population density and land cover as influential in many climate regions. On the other hand, importance of lagged anomalies of climatic features varied across models and generally had low relative magnitudes of importance.

Our use of baseline models of varying complexity allowed us to compare the relative performance of included models. While we did not identify a single “best” model for WNND prediction, NB models on the hexagonal or regional levels performed well overall and could be used as benchmark models. Predicted performance of model types across the United States was modulated by both the historical WNND patterns and variation in observed cases from these historical trends. Further work is needed to produce actionable predictions for early warning of WNND cases in the United States. Potential avenues include the use of more advanced machine learning architectures, improved prediction of emergence of cases, real‐time integration of vector surveillance data, utilization of additional surveillance systems, and more local models to guide public health and vector control activities.

## Conflict of Interest

The authors declare no conflicts of interest relevant to this study.

## Supporting information

Supporting Information S1

## Data Availability

All data are publicly available, as cited in the text and references. Human WNND case data are visible and downloadable on the CDC's West Nile virus Data & Maps webpage (Centers for Disease Control and Prevention, 2023). Demographic data from the 2010 census are openly available from the U.S. Census Bureau for download (United States Census Bureau, 2021). Monthly temperature (minimum and mean) and total precipitation data (1998–2021) are openly available from PRISM for download (PRISM Climate Group & Oregon State University, 2022). Land use data (2011) are openly available from the Multi‐Resolution Land Characteristics Consortium (MRLC) for download (U.S. Geological Survey, 2014). R statistical software and associated packages are openly available for download on CRAN and GitHub (Fritsch et al., 2019; Goodrich et al., 2022; Hyndman et al., 2022; Lenth, 2023; R Core Team, 2022; Redell, 2022; Stan Development Team, 2022; Walker, 2022). R scripts developed for data processing and analysis are openly available (Holcomb, 2023).
