# Supplementary material for: Multi‐Model Prediction of West Nile Virus Neuroinvasive Disease With Machine Learning for Identification of Important Regional Climatic Drivers
Source: Geohealth. 2023 Nov 17;7(11):e2023GH000906. doi: 10.1029/2023GH000906 (PMC10654557; doi:10.1029/2023GH000906)
Supplement: Supplementary file 1 — Supporting Information S1 [file GH2-7-e2023GH000906-s001.pdf]

GeoHealth

Supporting Information for

Multi-model prediction of West Nile virus neuroinvasive disease with machine learning for identification of important regional climatic drivers

Karen M. Holcomb[1,2], J. Erin Staples[2], Randall J. Nett[2], Charles B. Beard[2], Lyle R. Petersen[2], Stanley G. Benjamin[1,3], Benjamin W. Green[1,3], Hunter Jones[4], Michael A. Johansson[5]

[1]Global Systems Laboratory, National Oceanic and Atmospheric Administration, Boulder, CO. [2]Division of Vector-Borne Diseases, Centers for Disease Control and Prevention, Fort Collins, CO. [3]Cooperative Institute for Research in Environmental Sciences, University of Colorado Boulder, Boulder, CO. [4]Climate Prediction Office, National Oceanic and Atmospheric Administration, Silver Spring, MD. [5]Division of Vector-Borne Diseases, Centers for Disease Control and Prevention, San Juan, PR.

Contents of this file

Text S1
Figures S1 to S8
Tables S1 to S5

Introduction

The following text, figures, and tables provide additional, supporting details for the analysis and results described in the main text.

- Text S1 outlines additional details of model development and methods for converting bootstrap samples to probabilistic forecasts.
- Development and details of model fit for the autoregressive climate model (AR(1)-Climate) are also outlined in Text S1 and presented in Figures S1-S2 and Table S1.
- Figure S3 illustrates the mean logarithmic score of the Always Absent model in relation to the other ten models illustrated in Figure 2.
- Differences in the estimate marginal means of models relative to the NB-hex model by region are presented in Figure S4. Differences for all pairwise comparisons for which the 95% highest posterior density (HPD) interval between did not contain zero are included in Table S2. Differences in the estimated marginal means of models across regions for which the 95% HPD interval between did not contain zero are presented in Table S3.

- Variable importance for machine learning models fit with normalized anomalies in monthly climatic data is presented in Figure S5.
- Median importance of lagged variables in machine learning models fit with monthly climate anomalies is presented in Figure S6.
- Figure S7 illustrates the median proportion of summed connection weights positive vs. the median rank of variables from the seasonal and monthly neural network models. This provides a visual summary of the relative importance and direction of the relationship between covariates and number of WNND cases.
- A comparison of variable importance from random forest models fit with either raw or normalized anomalies in seasonal climate data presented in Figure S8. Table S4 compares the mean logarithmic score per climate region for these two models.
- Table S5 contains the top six important variables from the four machine learning models (RF-season, RF-month, NN-season, and NN-month).

Text S1.

Example of iterative model fitting and prediction

All models were iteratively trained and tested for prospectively predicting hexagon-annual WNND cases in 2015-2021. Predictions for each year were made with new models trained on all data prior to the prediction year of interest such that the training dataset included an additional year of data for predicting each following year. In other words, each type of model was trained on data through 2014 to predict WNND cases in 2015. New models were then trained on data that now included 2015, and these were used to predict cases in 2016. We continued this iterative procedure for annual predictions through 2021.

All models produce probabilistic predictions which were used for model evaluation (see Section 2.3 for details on the logarithmic score). Depending on the type of model, these were obtained from the fitted distribution (NB-hex, NB-region, and NB-nation models), prescribed by underlying assumptions of no cases (Always Absent naïve model), or bootstrap samples [AR(1), AR(1)-Climate, random forest (RF), and neural network (NN)]. These bootstrap samples incorporated both model (training residuals) and prediction uncertainty and were used to obtain probabilistic predictions (see next section for chosen method). See Section 2.2.1 for discussion of the negative binomial and Always Absent models.

Calculation of probabilistic forecast from bootstrapped samples

We considered two methods to produce probabilistic forecasts from modeling frameworks that predicted case counts (i.e., the AR(1), AR(1)-Climate, random forest, and neural network models). For the first method, we used the proportion of bootstrap samples equal to the number observed cases as the corresponding probability for calculating the logarithmic score. For the second method, we took 23 quantiles of the bootstrap samples (1%, 2.5%, 5%, 10%, 15%, ..., 85%, 90%, 95%, 97.5%, 99%) and fitted these to a negative binomial distribution. Then, using the fitted distribution, we calculated the probability corresponding to the observed number of cases. We selected the negative binomial distribution which minimized the squared difference between the quantiles of the sample and distribution. For each method, bootstrapped

samples were converted from the prediction scale (ln(cases+1)) to counts prior to calculating the proportions (Method 1) or quantiles (Method 2).

Similar logarithmic scores were obtained from both methods. Method 1 tended to produce lower scores (worse) than Method 2 because it did not produce smooth distributions across predicted case counts when not all outcomes in a particular range occurred in the bootstrapped samples. For these reasons, we chose to used Method 2 as our calculation method throughout.

Model fitting for negative binomial models

For both the NB-hex and NB-nation models, we used Stan models to perform MCMC sampling to fit the respective negative binomial distributions (i.e., estimate the mean (μ) and dispersion (φ) parameters) using normal (mean of zero and standard deviation of one) priors for μ and $\frac{1}{\sqrt{\varphi}}$. For all models, we ran four chains with a burn-in of 1,500 samples, and then collected an additional 1,500, resulting in 6,000 samples across the four chains. All models were checked for convergence. We used the median posterior estimate for the parameter values of the fitted negative binomial models.

Development of autoregressive climate model

To select the AR(1)-Climate model for each region, we fitted a series of 24 AR(1) models, each with a single exogenous climate covariate. We considered seasonal aggregations of anomalies in mean temperature and total precipitation for each season of the prediction year and previous year (i.e., winter, spring, summer, and fall) where Dec-Feb represented "winter", Mar-May represented "spring", Jun-Aug represented "summer", and "Sep-Nov" represented "fall". Seasonal anomalies were a subset of those calculated for use as covariates in the machine learning models (see Section 2.1 for details). As with the AR(1) model, we predicted the distribution of cases by generating 1,000 bootstrapped samples (sum of sample from prediction and randomly selected training residual) and fit a negative binomial model to the quantiles of the bootstrapped samples. We compared predictive accuracy of each candidate model across all hexes and predicted years (2015-2021) using logarithmic scoring to select the single best model which we designated as the AR(1)-Climate model. All models were fitted and predicted using the *Arima* function in the forecast package (Hyndman et al., 2022; Hyndman & Khandakar, 2008). See Figure S1 for the performance of each candidate model and region and Table S1 for the selected seasonal climate variable for each region. Figure S2 illustrates the fit coefficients for the AR(1)-Climate models.

The skill of the candidate models was very similar across prediction years with variation in the relative ranking by mean logarithmic score each year. Of the climate covariates selected in each climate region, we identified broad geographic trends. Broadly, anomalies in winter precipitation were selected in three contiguous climate regions (Southwest, South, and Ohio Valley) while anomalies in temperature were selected in the other six regions. More specifically, the anomaly in mean temperature during the previous spring (Mar-May) was selected in both the Upper Midwest and Northern Rockies and Plains regions while the anomaly in total precipitation in the previous winter (Dec-Feb) was selected in both the Ohio Valley and South regions. No other regions had the same climate covariate selected.

Cross-validation of random forest models

For the nested cross-validation of random forest models, we tried withholding either one- or two-year "windows" of training data. We did not observe significant differences between model fits, predictive performance, and important variables. For example, both window lengths resulted in similar in- and out-of-sample RMSE and MAE with variation across regions which window length minimized these metrics. We found small differences in the resulting mean logarithmic scores (range of difference ≤0.03).

Choice of climatic data

We chose to use normalized anomalies vs. the raw value of temperature and precipitation in models to reduce the correlation among variables and produce unbiases interpretation of the importance metrics from machine learning models. While machine learning models can produce robust predictions using highly correlated variables (Obite et al., 2020; Strobl et al., 2008), inclusion of these variables reduce the interpretability of importance metrics from the fitted model (Nicodemus & Malley, 2009). Since information is shared between such correlated variables, there is a reduction in apparent importance of these variables. As one of our goals was to identify highly important factors for prediction, we chose to use normalized anomalies.

To compare how this decision may have impacted our conclusions, we fitted an additional random forest (RF) model. For this model, we used seasonal mean temperature and total precipitation instead of normalized anomalies. All other variables were the same as the RF-season model (see Section 2.2.2 for details). We chose the RF-season model because it was identified as the "best" of the machine learning models overall (see Figure 3).

We did find some variation in the magnitude of variable importance between the RF-season model using climate anomalies vs. raw climate values (Figure S8). Across regions, variables for precipitation often had higher importance in the model with raw climate values than with normalized anomalies. In contrast, lagged WNND cases tended to have higher importance when climate anomalies were included. Overall, we estimated very similar mean logarithmic scores (Table S4), indicating similar predictive accuracy of the two model versions.

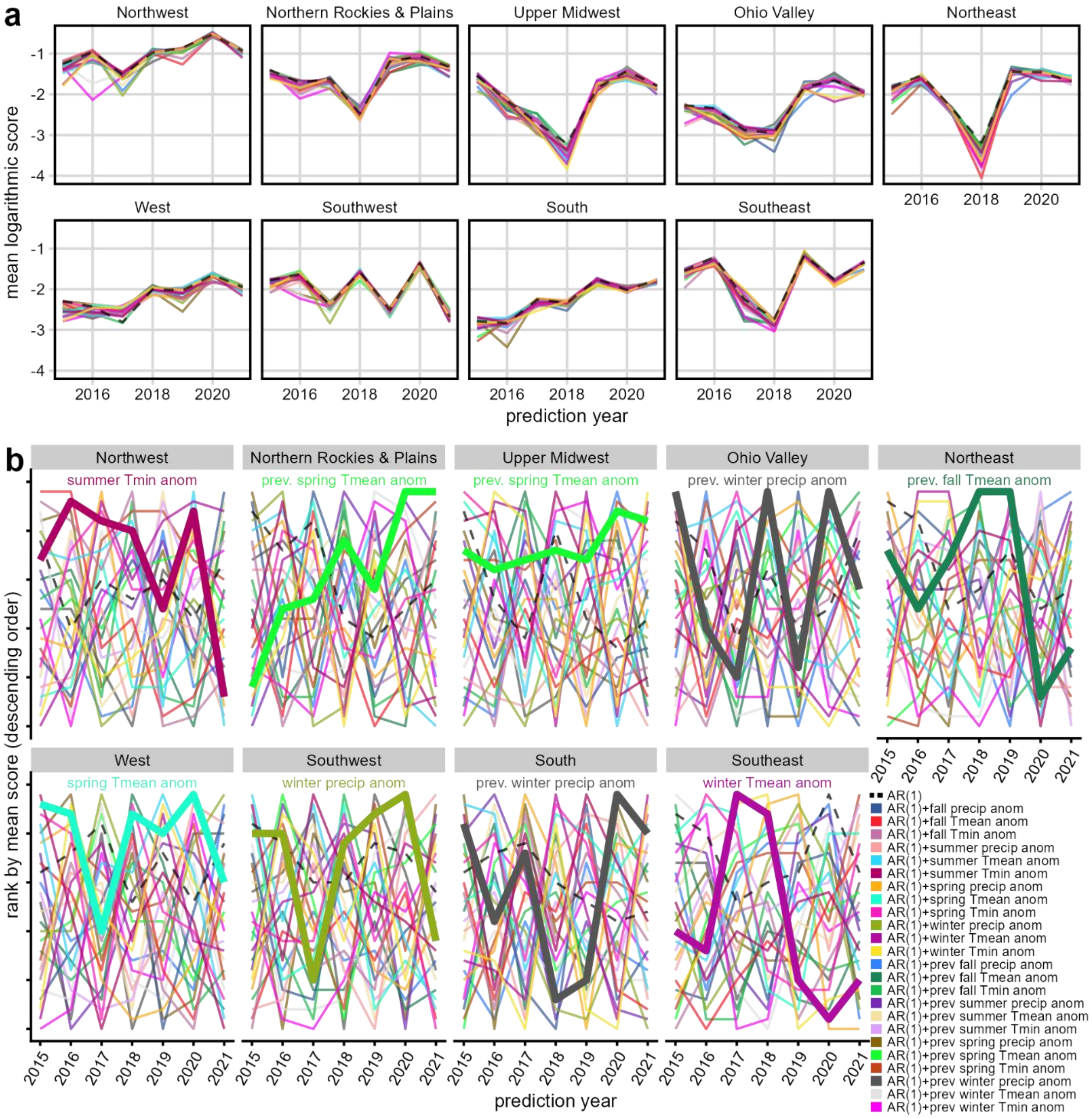

Figure S1. Mean logarithmic score of AR(1) and AR(1)-Climate candidate models per climate region. a) Mean logarithmic score per year (2015-2021) of AR(1) and AR(1)-Climate candidate models. Smaller scores (closer to 0) indicate better scores. b) Ranking of models based on mean logarithmic score by prediction year (descending rank). Text inset indicates climate covariate in the candidate model with the lowest mean logarithmic score overall (i.e., selected AR(1)-Climate model per region (bold line), see Table S1 and Figure S2 for further details). Order of panels represent the relative geographic location of the nine climate regions (see Figure 1a). Seasons were defined as three-month aggregations of normalized anomalies in winter (Dec-Feb), spring (Mar-May), summer (Jun-Aug), and fall (Sep-Nov). Tmin: minimum temperature, Tmean: mean temperature, Precip: total precipitation, anom: normalized anomaly

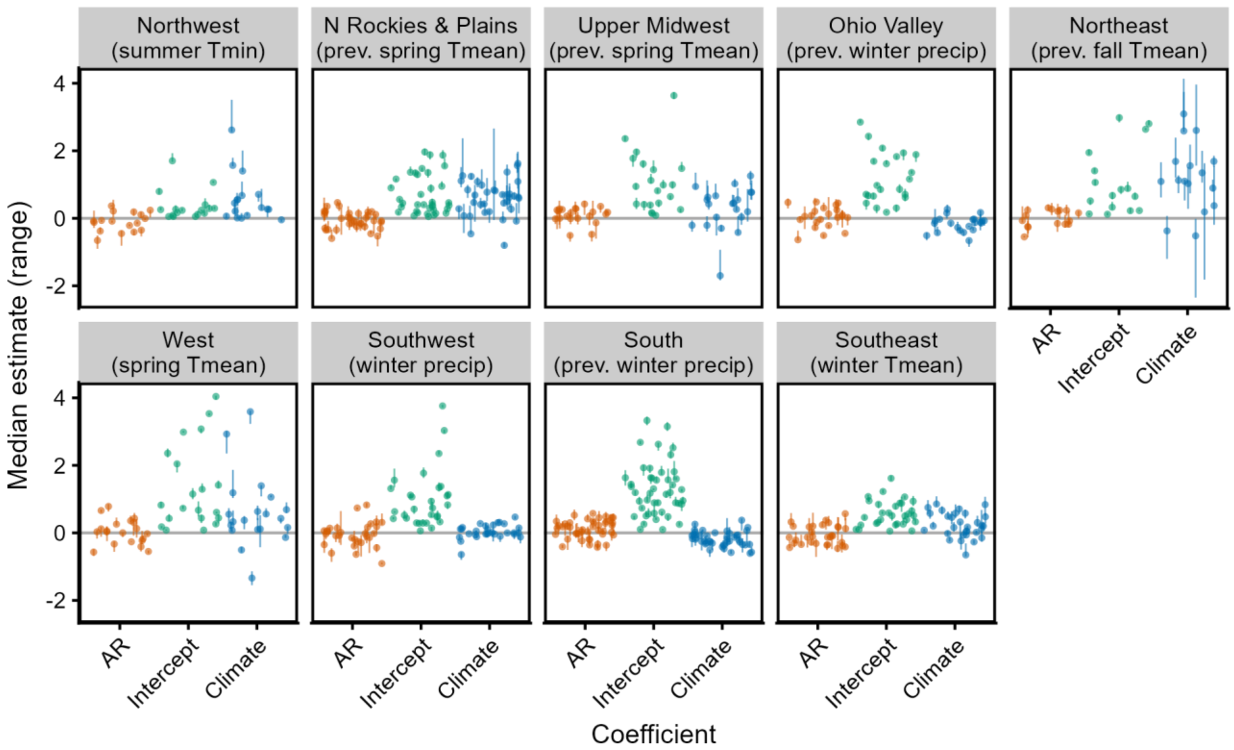

Figure S2. Coefficients from unique AR(1)-Climate models fit per hexagon and prediction year (2015-2021). Median (dot) and range (line) of coefficients [intercept, autoregressive (AR) and regression (climate variable)] summarized per hexagon. Model intercept corresponds to mean number of WNND cases (natural log scale). Order of panels represent the relative geographic location of the nine climate regions in the US (see Figure 1a) and subheadings indicate the normalized anomaly in the seasonal climate variable used per region (i.e., "best" covariate; see Figure S1). Seasons were defined as three-month aggregations for winter (Dec-Feb), spring (Mar-May), summer (Jun-Aug), and fall (Sep-Nov). Tmin: minimum temperature, Tmean: mean temperature, Precip: total precipitation.

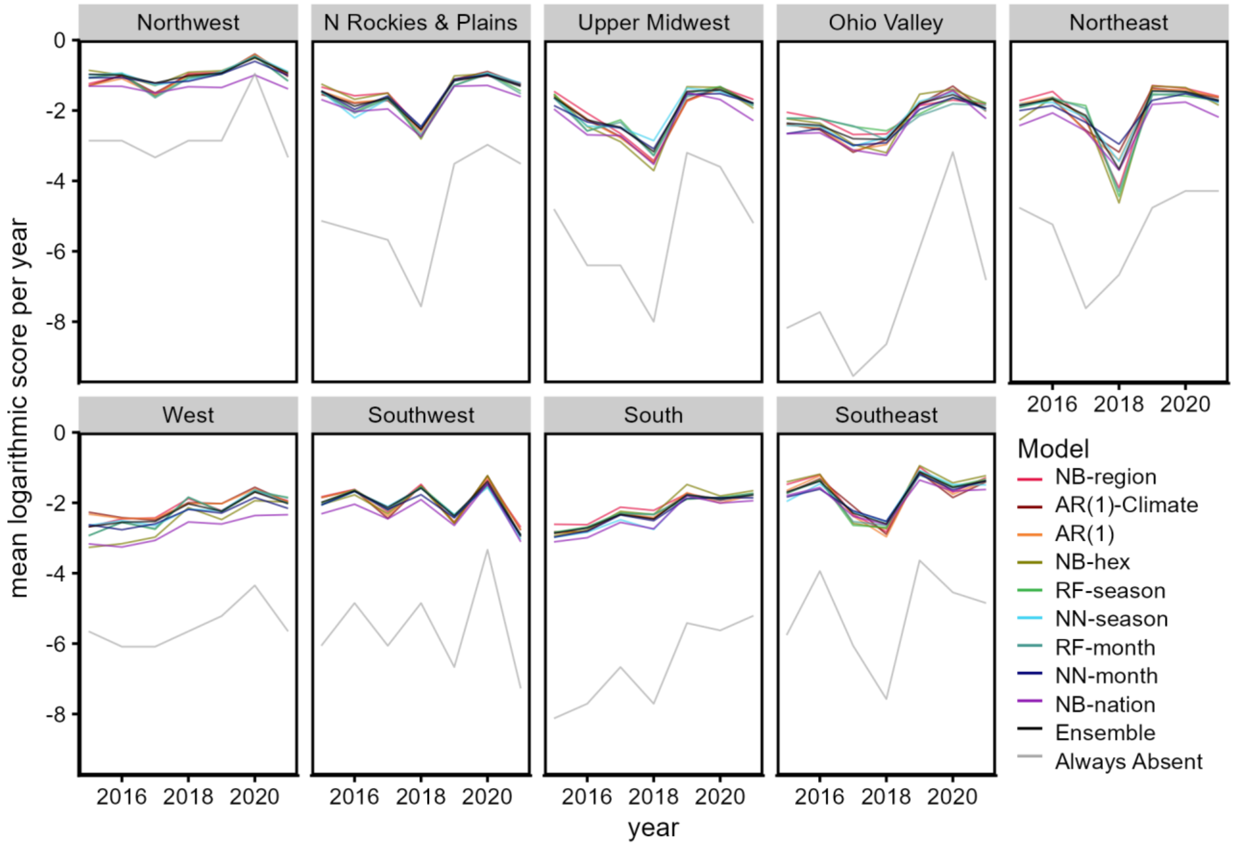

Figure S3. Mean yearly logarithmic score of models for WNND prediction. Scores closer to zero indicate better performance. Order of panels represent the relative geographic location of the nine climate regions in the US (see Figure 1a).

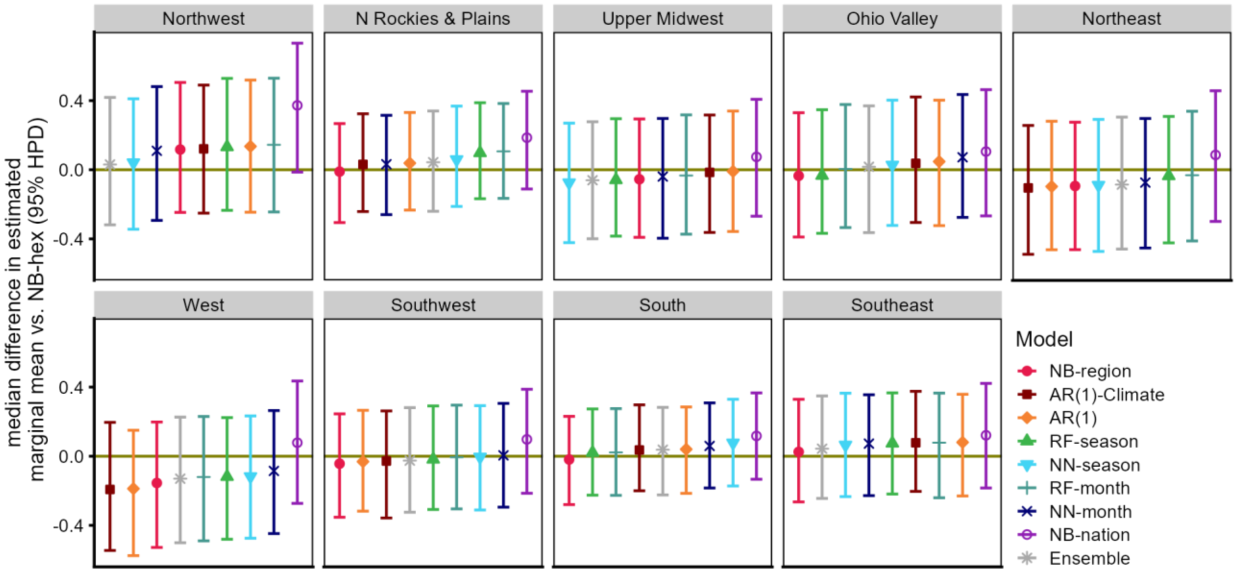

Figure S4. Median difference in estimated marginal means of predicted surprisal for models relative to the NB-hex model by climate region. Range indicates 95% highest probability density (HPD) of the difference. Comparison with Always Absent model omitted for scale (see Table S2). Increasing values of surprisal indicate worse forecast skill.

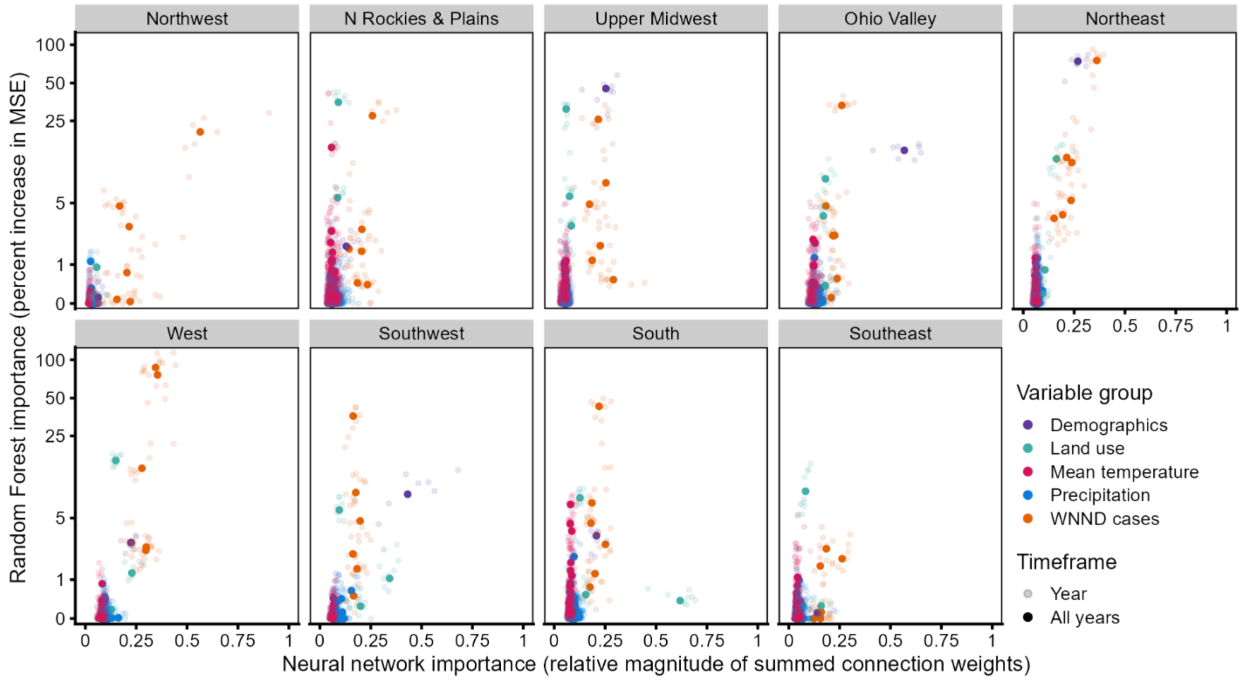

Figure S5. Variable importance for machine learning models fit with normalized anomalies in monthly climatic data. Random forest importance (y-axes) measured as median percent increase in mean squared prediction error (MSE) when variable permuted. Neural network importance (x-axes) measured as median relative magnitude of summed connection weights. See Figure S6 and Table S5 for further details on variable importance. Order of panels represent the relative geographic location of the nine climate regions in the US (see Figure 1a).

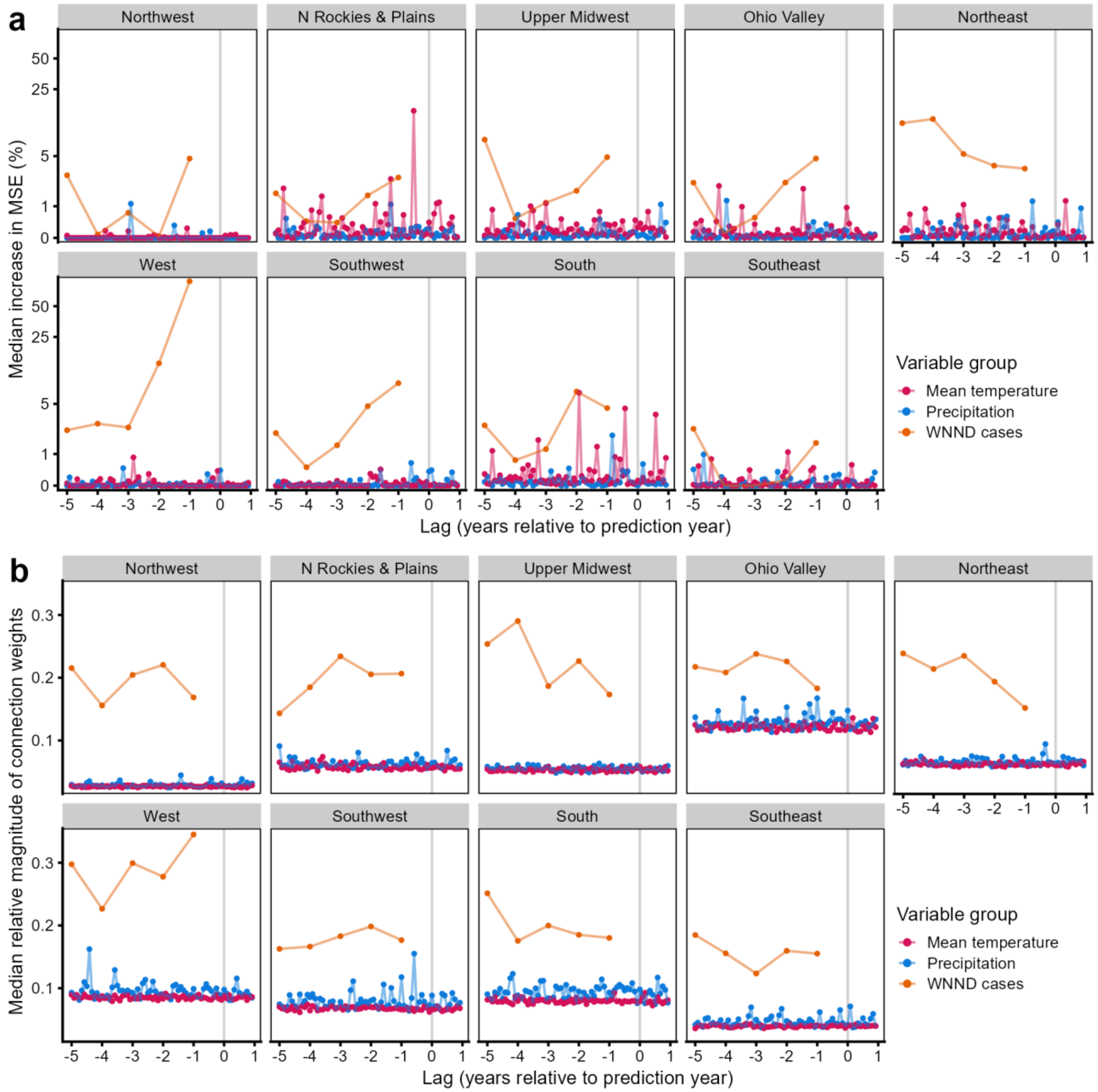

Figure S6. Median importance of lagged variables in models fit with monthly climate data (normalized anomalies). Importance based on a) median increase in prediction error (mean squared error, MSE) when variable permuted in random forest or b) median relative magnitude of summed connection weights in neural network. Importance measured across all models and prediction years. Lags of zero-years indicate values from prediction year. See Table S5 for further details on variable importance. Order of panels represent the relative geographic location of the nine climate regions in the US (see Figure 1a).

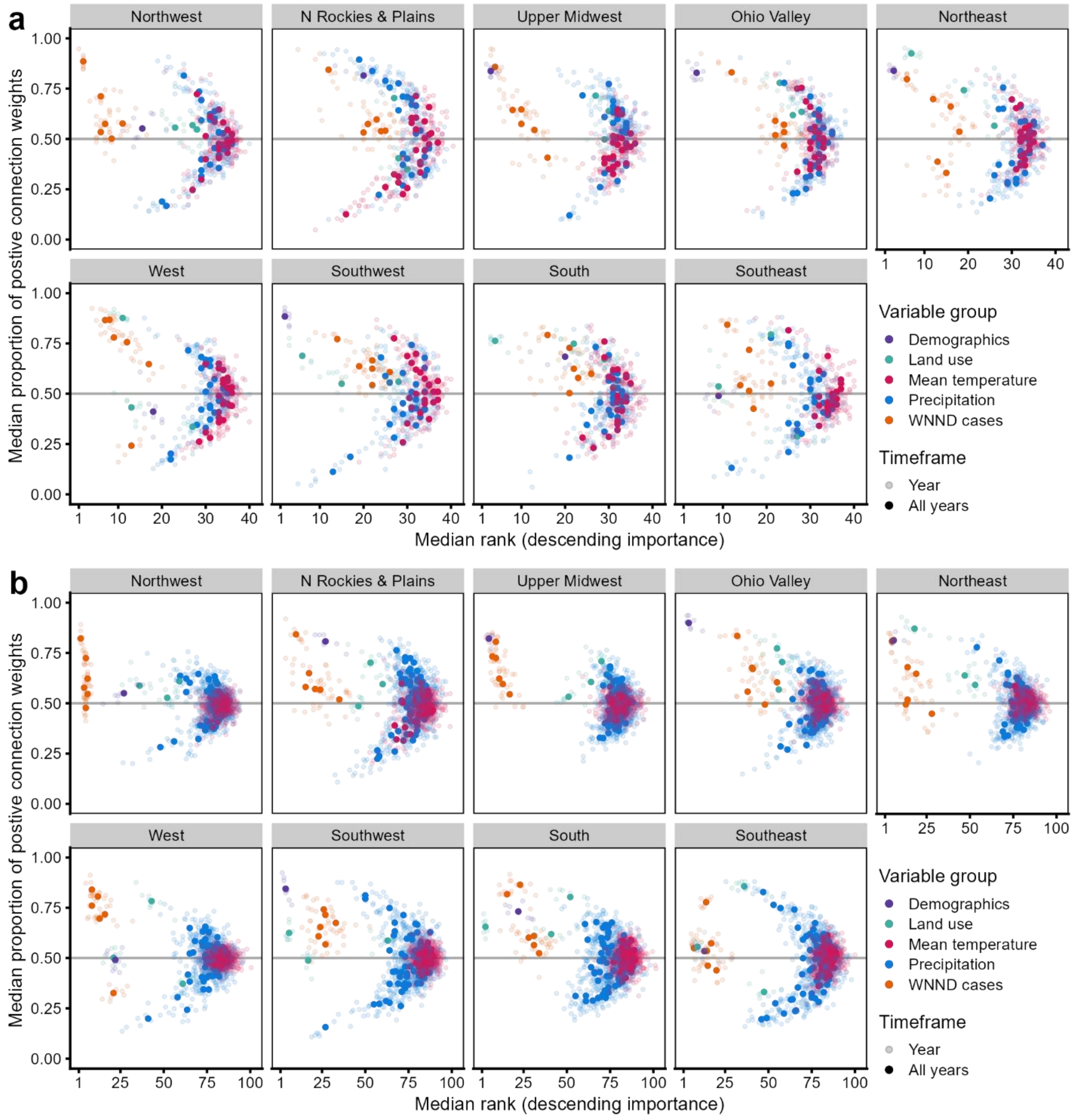

Figure S7. Median proportion of summed connection weights positive from neural networks. Neural networks fit with a) seasonal or b) monthly climate data. Variables ranked by median relative magnitude of summed connection weights (see Figure 4). Points above horizontal line indicate most summed connection weights were positive while points below the line indicate most summed connection weights were negative. See Table S5 for further details on variable importance. Order of panels represent the relative geographic location of the nine climate regions in the US (see Figure 1a).

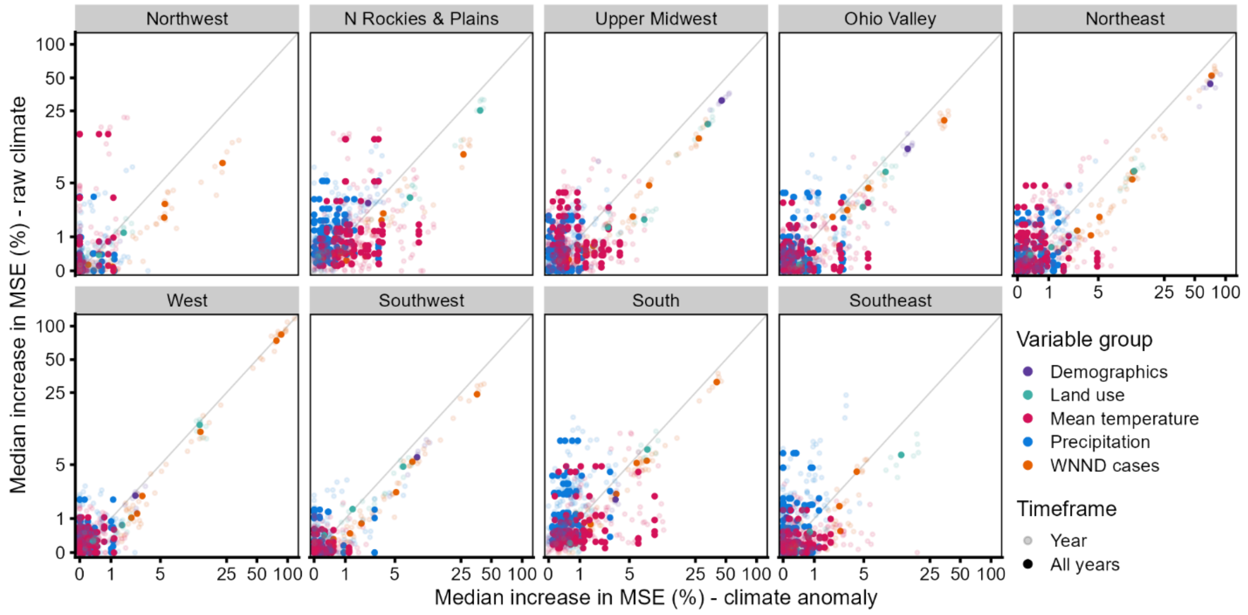

Figure S8. Importance of variables from random forests fitted with raw or normalized anomalies of seasonal climate data. Importance measured as median increase in mean squared error (MSE) when the variable was permuted. Diagonal grey line indicates 1:1 correspondence between importance of fitted models. Order of panels represent the relative geographic location of the nine climate regions in the US (see Figure 1a).

Table S1. Climate variable selected for AR(1)-Climate model per climate region. Normalized anomalies calculated by season using three-month aggregations. Winter: Dec-Feb; Spring: Mar-May; Summer: Jun-Aug; Fall: Sep-Nov.

**Climate Region**	**Climate variable**†
Northwest	Summer minimum temperature anomaly
West	Spring mean temperature anomaly
Southwest	Winter total precipitation anomaly
Northern Rockies & Plains	Previous spring mean temperature anomaly
South	Previous winter total precipitation anomaly
Ohio Valley	Previous winter total precipitation anomaly
Upper Midwest	Previous spring mean temperature anomaly
Northeast	Previous fall mean temperature anomaly
Southeast	Winter mean temperature anomaly

† *Normalized anomaly (number of standard deviations from long-term average) in indicated seasonal climatic variable. Long-term average calculated for 2000-2021.*

Table S2. Difference in estimated marginal means for models in each climate region. Only contrasts between models where the 95% highest posterior density (HPD) interval did not contain zero are included. Negative differences indicate worse relative forecast skill of the second model in the contrast.

Climate Region	Contrast	Median difference	Lower HPD	Upper HPD
Northwest	Ensemble - Always Absent	-1.067	-1.461	-0.686
	AR(1) - Always Absent	-0.963	-1.334	-0.579
	AR(1)-Climate - Always Absent	-0.973	-1.374	-0.591
	NN-month - Always Absent	-0.990	-1.362	-0.602
	NN-season - Always Absent	-1.060	-1.442	-0.704
	RF-month - Always Absent	-0.957	-1.330	-0.561
	RF-season - Always Absent	-0.963	-1.357	-0.584
	NB-hex - Always Absent	-1.100	-1.495	-0.725
	NB-region - Always Absent	-0.980	-1.363	-0.582
	NB-nation - Always Absent	-0.730	-1.116	-0.361
West	Ensemble - Always Absent	-0.901	-1.263	-0.524
	AR(1) - Always Absent	-0.957	-1.297	-0.569
	AR(1)-Climate - Always Absent	-0.964	-1.319	-0.597
	NN-month - Always Absent	-0.856	-1.192	-0.474
	NN-season - Always Absent	-0.889	-1.288	-0.552
	RF-month - Always Absent	-0.893	-1.254	-0.531
	RF-season - Always Absent	-0.891	-1.241	-0.525
	NB-hex - Always Absent	-0.770	-1.160	-0.425
	NB-region - Always Absent	-0.925	-1.263	-0.576
	NB-nation - Always Absent	-0.690	-1.060	-0.326
Southwest	Ensemble - Always Absent	-1.023	-1.321	-0.734
	AR(1) - Always Absent	-1.027	-1.316	-0.736
	AR(1)-Climate - Always Absent	-1.026	-1.343	-0.724
	NN-month - Always Absent	-0.992	-1.282	-0.693
	NN-season - Always Absent	-1.000	-1.306	-0.718
	RF-month - Always Absent	-1.000	-1.306	-0.715
	RF-season - Always Absent	-1.014	-1.328	-0.726
	NB-hex - Always Absent	-0.996	-1.284	-0.694
	NB-region - Always Absent	-1.041	-1.329	-0.742
	NB-nation - Always Absent	-0.897	-1.199	-0.610
Northern Rockies and Plains	Ensemble - Always Absent	-1.125	-1.396	-0.826
	AR(1) - Always Absent	-1.132	-1.436	-0.866
	AR(1)-Climate - Always Absent	-1.139	-1.408	-0.849
	NN-month - Always Absent	-1.137	-1.410	-0.843
	NN-season - Always Absent	-1.111	-1.390	-0.823

	RF-month - Always Absent	-1.064	-1.348	-0.801
	RF-season - Always Absent	-1.068	-1.359	-0.781
	NB-hex - Always Absent	-1.166	-1.452	-0.885
	NB-region - Always Absent	-1.185	-1.478	-0.908
	NB-nation - Always Absent	-0.982	-1.283	-0.706
South	Ensemble - Always Absent	-1.075	-1.324	-0.821
	AR(1) - Always Absent	-1.072	-1.310	-0.830
	AR(1)-Climate - Always Absent	-1.076	-1.321	-0.834
	NN-month - Always Absent	-1.054	-1.301	-0.810
	NN-season - Always Absent	-1.034	-1.285	-0.796
	RF-month - Always Absent	-1.093	-1.336	-0.852
	RF-season - Always Absent	-1.093	-1.337	-0.848
	NB-hex - Always Absent	-1.112	-1.361	-0.868
	NB-region - Always Absent	-1.135	-1.368	-0.873
	NB-nation - Always Absent	-0.996	-1.248	-0.747
Ohio Valley	Ensemble - Always Absent	-1.157	-1.534	-0.792
	AR(1) - Always Absent	-1.126	-1.484	-0.764
	AR(1)-Climate - Always Absent	-1.131	-1.497	-0.780
	NN-month - Always Absent	-1.099	-1.479	-0.740
	NN-season - Always Absent	-1.145	-1.501	-0.762
	RF-month - Always Absent	-1.168	-1.527	-0.801
	RF-season - Always Absent	-1.202	-1.560	-0.830
	NB-hex - Always Absent	-1.170	-1.540	-0.811
	NB-region - Always Absent	-1.206	-1.556	-0.847
	NB-nation - Always Absent	-1.067	-1.427	-0.685
Upper Midwest	Ensemble - Always Absent	-0.970	-1.314	-0.628
	AR(1) - Always Absent	-0.917	-1.250	-0.570
	AR(1)-Climate - Always Absent	-0.926	-1.256	-0.577
	NN-month - Always Absent	-0.947	-1.285	-0.591
	NN-season - Always Absent	-0.983	-1.348	-0.652
	RF-month - Always Absent	-0.941	-1.281	-0.597
	RF-season - Always Absent	-0.968	-1.317	-0.623
	NB-hex - Always Absent	-0.907	-1.256	-0.548
	NB-region - Always Absent	-0.963	-1.312	-0.629
	NB-nation - Always Absent	-0.833	-1.198	-0.508
Northeast	Ensemble - Always Absent	-0.986	-1.364	-0.589
	AR(1) - Always Absent	-0.999	-1.383	-0.605
	AR(1)-Climate - Always Absent	-1.008	-1.395	-0.638
	NN-month - Always Absent	-0.980	-1.361	-0.600
	NN-season - Always Absent	-0.996	-1.382	-0.624
	RF-month - Always Absent	-0.946	-1.323	-0.559

	RF-season - Always Absent	-0.938	-1.321	-0.595
	NB-hex - Always Absent	-0.901	-1.293	-0.540
	NB-region - Always Absent	-0.994	-1.400	-0.620
	NB-nation - Always Absent	-0.820	-1.187	-0.437
Southeast	Ensemble - Always Absent	-1.105	-1.404	-0.794
	AR(1) - Always Absent	-1.071	-1.370	-0.766
	AR(1)-Climate - Always Absent	-1.071	-1.372	-0.786
	NN-month - Always Absent	-1.076	-1.369	-0.765
	NN-season - Always Absent	-1.086	-1.394	-0.796
	RF-month - Always Absent	-1.071	-1.370	-0.783
	RF-season - Always Absent	-1.077	-1.369	-0.775
	NB-hex - Always Absent	-1.150	-1.464	-0.856
	NB-region - Always Absent	-1.124	-1.427	-0.823
	NB-nation - Always Absent	-1.027	-1.332	-0.730

Table S3. Difference in estimated marginal means for models across all nine climate regions. Only contrasts between models where the 95% highest posterior density (HPD) interval did not contain zero are included. Negative differences indicate worse relative forecast skill of the second model in the contrast.

Contrast	Median difference	Lower HPD	Upper HPD
Ensemble - NB-nation	-0.133	-0.250	-0.031
AR(1) - NB-nation	-0.123	-0.226	-0.014
AR(1)-Climate - NB-nation	-0.128	-0.235	-0.027
NN-season - NB-nation	-0.116	-0.223	-0.007
RF-month - NB-nation	-0.109	-0.217	-0.002
RF-season - NB-nation	-0.119	-0.229	-0.010
NB-hex - NB-nation	-0.121	-0.227	-0.011
NB-region - NB-nation	-0.159	-0.265	-0.055
Ensemble - Always Absent	-1.054	-1.160	-0.942
AR(1) - Always Absent	-1.044	-1.149	-0.940
AR(1)-Climate - Always Absent	-1.048	-1.151	-0.941
NN-month - Always Absent	-1.024	-1.130	-0.913
NN-season - Always Absent	-1.036	-1.144	-0.934
RF-month - Always Absent	-1.030	-1.135	-0.924
RF-season - Always Absent	-1.040	-1.146	-0.939
NB-hex - Always Absent	-1.040	-1.140	-0.926
NB-region - Always Absent	-1.080	-1.180	-0.971
NB-nation - Always Absent	-0.921	-1.030	-0.815

Table S4. Mean logarithmic score for WNND prediction from random forest models fitted with either raw or normalized anomalies in seasonal climate data. Scores closer to 0 indicate better performance.

Climate region	Mean logarithmic score – raw climate	Mean logarithmic score – climate anomalies
Northwest	-1.01	-0.98
West	-2.21	-2.21
Southwest	-2.03	-2.04
Northern Rockies and Plains	-1.64	-1.59
South	-2.17	-2.20
Ohio Valley	-2.12	-2.12
Upper Midwest	-2.03	-2.04
Northeast	-2.11	-2.12
Southeast	-1.75	-1.67

Table S5. Top six variables per climate region. Random forest (RF) and neural network (NN) models trained with either lagged seasonal[†] or monthly climate data (normalized anomalies, lagged 0-5 years relative to the prediction year).

Climate Region	Rank[‡]	RF-season		NN-season		RF-month		NN-month	
		Variable	IncMSE[#]	Variable	CW[+]	Variable	IncMSE[#]	Variable	CW[+]
Northwest	1	Median historic cases	0.24	Median historic cases	0.73	Median historic cases	0.2	Median historic cases	0.56
	2	Cases (1-yr lag)	0.06	Cases (2-yr lag)	0.17	Cases (1-yr lag)	0.05	Cases (2-yr lag)	0.22
	3	Cases (5-yr lag)	0.05	Cases (5-yr lag)	0.16	Cases (5-yr lag)	0.03	Cases (3-yr lag)	0.2
	4	Crops	0.01	Cases (3-yr lag)	0.13	Crops	0.01	Cases (5-yr lag)	0.22
	5	Spring mean temp (1-yr lag)	0.01	Cases (1-yr lag)	0.11	Feb precip (3-yr lag)	0.01	Cases (1-yr lag)	0.17
	6	Spring precip (3-yr lag)	0.01	Cases (4-yr lag)	0.11	Cases (3-yr lag)	0.01	Cases (4-yr lag)	0.16
West	1	Cases (1-yr lag)	0.85	Median historic cases	0.38	Cases (1-yr lag)	0.87	Cases (1-yr lag)	0.34
	2	Median historic cases	0.77	Cases (5-yr lag)	0.38	Median historic cases	0.76	Median historic cases	0.35
	3	Cases (2-yr lag)	0.14	Cases (1-yr lag)	0.34	Cases (2-yr lag)	0.14	Cases (5-yr lag)	0.3
	4	Urban	0.14	Urban	0.29	Urban	0.16	Cases (2-yr lag)	0.28
	5	Cases (4-yr lag)	0.03	Cases (3-yr lag)	0.3	Cases (4-yr lag)	0.03	Cases (3-yr lag)	0.3

	6	Cases (5-yr lag)	0.03	Crops	0.27	Population density	0.03	Crops	0.23
Southwest	1	Median historic cases	0.33	Population density	0.65	Median historic cases	0.36	Population density	0.43
	2	Cases (1-yr lag)	0.08	Crops	0.4	Cases (1-yr lag)	0.08	Crops	0.34
	3	Population density	0.08	Fall precip (4-yr lag)	0.22	Population density	0.08	Wetlands	0.2
	4	Urban	0.07	Median historic cases	0.21	Urban	0.06	Cases (2-yr lag)	0.2
	5	Cases (2-yr lag)	0.05	Wetlands	0.22	Cases (2-yr lag)	0.05	Cases (3-yr lag)	0.18
	6	Summer precip (1-yr lag)	0.03	Fall precip (2-yr lag)	0.19	Cases (5-yr lag)	0.02	Median historic cases	0.16
Northern Rockies and Plains	1	Urban	0.37	Median historic cases	0.35	Urban	0.35	Median historic cases	0.26
	2	Median historic cases	0.25	Winter mean temp (3-yr lag)	0.29	Median historic cases	0.27	Cases (3-yr lag)	0.23
	3	Summer mean temp (4-year lag)	0.1	Spring precip (0-yr lag)	0.26	Jul mean temp (1-yr lag)	0.15	Cases (2-yr lag)	0.21
	4	Crops	0.07	Cases (3-yr lag)	0.26	Crops	0.06	Cases (1-yr lag)	0.21
	5	Cases (1-yr lag)	0.03	Population density	0.24	Cases (1-yr lag)	0.03	Cases (4-yr lag)	0.18

	6	Cases (5-yr lag)	0.03	Cases (2-yr lag)	0.23	Oct mean temp (2-yr lag)	0.03	Population density	0.13
South	1	Median historic cases	0.4	Wetlands	0.51	Median historic cases	0.43	Wetlands	0.62
	2	Winter mean temp (2-yr lag)	0.1	Cases (5-yr lag)	0.23	Urban	0.08	Cases (5-yr lag)	0.25
	3	Cases (2-yr lag)	0.08	Population density	0.2	Cases (2-yr lag)	0.07	Population density	0.21
	4	Urban	0.08	Cases (4-yr lag)	0.19	Feb mean temp (2-yr lag)	0.07	Median historic cases	0.22
	5	Summer mean temp (1-yr lag)	0.06	Median historic cases	0.2	Cases (1-yr lag)	0.04	Cases (3-yr lag)	0.2
	6	Cases (1-yr lag)	0.06	Spring precip (1-yr lag)	0.19	Aug mean temp (1-yr lag)	0.04	Cases (1-yr lag)	0.18
Ohio Valley	1	Median historic cases	0.33	Population density	0.66	Median historic cases	0.33	Population density	0.57
	2	Population density	0.16	Median historic cases	0.34	Population density	0.14	Median historic cases	0.26
	3	Urban	0.09	Cases (3-yr lag)	0.23	Urban	0.08	Cases (3-yr lag)	0.24
	4	Spring mean temp (4-yr lag)	0.06	Cases (5-yr lag)	0.22	Cases (1-yr lag)	0.05	Cases (5-yr lag)	0.22

	5	Crops	0.05	Crops	0.22	Crops	0.04	Cases (2-yr lag)	0.23
	6	Cases (1-yr lag)	0.05	Cases (1-yr lag)	0.21	Cases (2-yr lag)	0.02	Cases (4-yr lag)	0.21
Upper Midwest	1	Population density	0.45	Population density	0.42	Population density	0.45	Population density	0.25
	2	Urban	0.32	Median historic cases	0.38	Urban	0.31	Cases (4-yr lag)	0.29
	3	Median historic cases	0.27	Cases (5-yr lag)	0.27	Median historic cases	0.26	Cases (5-yr lag)	0.25
	4	Cases (5-yr lag)	0.08	Cases (3-yr lag)	0.22	Crops	0.06	Median historic cases	0.22
	5	Crops	0.07	Cases (4-yr lag)	0.27	Cases (5-yr lag)	0.08	Cases (2-yr lag)	0.23
	6	Cases (1-yr lag)	0.05	Cases (2-yr lag)	0.19	Cases (1-yr lag)	0.05	Cases (3-yr lag)	0.19
Northeast	1	Population density	0.73	Population density	0.58	Median historic cases	0.75	Median historic cases	0.36
	2	Median historic cases	0.72	Median historic cases	0.34	Population density	0.74	Population density	0.27
	3	Cases (4-yr lag)	0.11	Urban	0.29	Cases (4-yr lag)	0.13	Cases (5-yr lag)	0.24
	4	Cases (5-yr lag)	0.12	Cases (3-yr lag)	0.22	Cases (5-yr lag)	0.11	Cases (3-yr lag)	0.23
	5	Urban	0.11	Cases (5-yr lag)	0.22	Urban	0.12	Cases (4-yr lag)	0.21
	6	Cases (3-yr lag)	0.04	Cases (1-yr lag)	0.19	Cases (3-yr lag)	0.05	Urban	0.16

Southeast	1	Urban	0.13	Population density	0.3	Urban	0.09	Median historic cases	0.26
	2	Cases (5-yr lag)	0.04	Wetlands	0.3	Cases (5-yr lag)	0.02	Wetlands	0.16
	3	Cases (1-yr lag)	0.03	Cases (1-yr lag)	0.26	Median historic cases	0.02	Population density	0.14
	4	Median historic cases	0.02	Fall precip (1-yr lag)	0.23	Cases (1-yr lag)	0.02	Cases (1-yr lag)	0.16
	5	Spring mean temp (0-yr lag)	0.02	Median historic cases	0.23	Feb mean temp (2-yr lag)	0.01	Cases (2-yr lag)	0.16
	6	Fall mean temp (2-yr lag)	0.02	Cases (2-yr lag)	0.21	May precip (5-yr lag)	0.01	Cases (5-yr lag)	0.18

† Seasons represented by three-month aggregations: winter (Dec-Feb), spring (Mar-May), summer (Jun-Aug), and fall (Sep-Nov).

‡ Variable rank by median order of importance across all models. In each model, variables ranked by median relative importance (RF: increase in mean squared error; NN: relative magnitude of summed connection weights). In a few cases, median relative importance is not decreasing with decreasing rank.

# Median increase in mean squared error (MSE) of prediction when variable permuted.

+ Median relative magnitude (0-1) of summed connection weights.

Precip: total precipitation; Temp: temperature; Cases: WNND cases
